# Methyl jasmonate elicitation increases vitexin production in protocorm-like body-derived cultures of *Grammatophyllum speciosum* Blume

**DOI:** 10.3389/fpls.2026.1915401

**Published:** 2026-10-28

**Authors:** Rittipun Rungruang, Wittawat Ratanathavorn, Tasanee Panichakul, Sasithorn Kongruang

**Affiliations:** 1 Department of Cosmetic Science, Faculty of Science and Technology, Suan Dusit University, Bangkok, Thailand; 2 Department of Biotechnology, Faculty of Applied Science, King Mongkut’s University of Technology North Bangkok, Bangkok, Thailand

**Keywords:** antioxidant activity, C-glycosylflavone, enzyme inhibition, orchid tissue culture, phenylalanine ammonia-lyase, phenylpropanoid pathway

## Abstract

*Grammatophyllum speciosum* Blume (Orchidaceae) is a giant orchid that accumulates phenolic glucosides and C-glycosylflavones, including the bioactive flavone vitexin. Vitexin is a pharmaceutically and cosmetically valuable compound obtained mainly from aerial tissues of wild-harvested plants, motivating controlled *in vitro* production. Methyl jasmonate (MeJA) is a widely used elicitor of phenylpropanoid and flavonoid biosynthesis; however, its effects on vitexin accumulation in protocorm-like bodies (PLBs) of *G. speciosum* in liquid culture systems have not yet been investigated. This study aimed to establish a PLB-derived liquid culture of *G. speciosum* and to evaluate the effects of MeJA (0, 50, 75, and 100 µM) on culture growth, phenolic and flavonoid accumulation, vitexin production, phenylalanine ammonia-lyase (PAL) activity, antioxidant capacity, and enzyme inhibitory activities. The cultures showed a sigmoidal growth profile with the highest dry growth index (DGI, 0.72 ± 0.09) on Day 32. MeJA elicitation for 12 days reduced biomass in a concentration-dependent manner (DGI 0.066 ± 0.007 to 0.050 ± 0.001 at Day 12 versus 0.089 ± 0.002 in untreated cultures), whereas the PLB clusters remained morphologically intact. Treatment with 75 µM MeJA gave the highest total phenolic (14.267 ± 0.273 mg GAE/g CDW) and total flavonoid (27.093 ± 0.504 mg QE/g CDW) contents. Vitexin was quantified by HPLC-DAD and its identity confirmed by LC-ESI-QTOF-MS; cultures treated with 75 µM MeJA contained 420.05 ± 9.96 µg vitexin/g culture dry weight, approximately twofold higher than untreated controls (208.33 ± 2.69 µg/g CDW). PAL activity was rapidly and transiently induced, peaking 12 h after 75 µM MeJA treatment. The same treatment also produced the highest DPPH (5.30 ± 0.20 mg TE/g CDW) and ABTS (10.64 ± 0.25 mg TE/g CDW) radical-scavenging and copper-chelating (6.54 ± 0.29 mg EDTA/g CDW) activities, and the strongest inhibition of collagenase (55.62 ± 0.34%), elastase (42.40 ± 0.63%), and tyrosinase (36.01 ± 1.71%). These results indicate that MeJA is associated with activation of the phenylpropanoid pathway and enhanced vitexin, phenolic, and flavonoid accumulation in *G. speciosum*. Overall, MeJA-elicited PLB-derived liquid cultures represent a controlled *in vitro* system for producing vitexin-containing extracts with antioxidant and enzyme-inhibitory activities.

## 1 Introduction

*Grammatophyllum speciosum* Blume, commonly known as the giant or tiger orchid, is among the largest orchid species worldwide and is distributed across tropical Southeast Asia, including Thailand, Malaysia, and Indonesia. In Thai traditional medicine, *G. speciosum* is used as a remedy for fever, insect bites, skin rashes, and inflammation, and its extracts have been reported to possess antioxidant, anti-aging, anti-collagenase, skin-brightening, and anticancer activities (Thamrongwatwongsa et al., 2024). These bioactivities are attributed to a diverse range of secondary metabolites, including the phenolic glucoside gastrodin and the C-glycosylflavones vitexin and orientin, which have been identified as the principal bioactive constituents of this species (Yingchutrakul et al., 2021). Plant secondary metabolites, including phenolics, flavonoids, and alkaloids, are synthesized through complex biosynthetic pathways and play important roles in plant defense and human health (Rao and Ravishankar, 2002). Among these pathways, the phenylpropanoid pathway is of central importance because it provides the C6-C3 backbone for the biosynthesis of phenolic compounds and feeds into flavonoid biosynthesis via 4-coumaroyl-CoA, supplying substrates for C-glycosylflavones, such as vitexin (Fraser and Chapple, 2011). The entry enzyme of this pathway, phenylalanine ammonia-lyase (PAL), catalyzes the conversion of L-phenylalanine to trans-cinnamic acid, and is widely regarded as a key regulatory node controlling carbon flux into phenylpropanoid and flavonoid metabolism (Fraser and Chapple, 2011). Vitexin (apigenin-8-C-β-D-glucopyranoside) is a bioactive C-glycosylflavone formed by C-glycosyltransferase-mediated glycosylation of an apigenin-derived intermediate (typically 2-hydroxynaringenin), producing a glycosidic bond that is resistant to enzymatic and acidic hydrolysis and that contributes to its metabolic stability *in vivo*. Vitexin has well-documented antioxidant, anti-inflammatory, anticancer, and neuroprotective properties, and has attracted increasing attention for pharmaceutical and cosmetic applications (Babaei et al., 2020; Thamrongwatwongsa et al., 2024). Previous phytochemical investigations of *G. speciosum* have reported vitexin mainly in aerial tissues, including leaves and pseudobulbs; however, the capacity of this species to produce vitexin under controlled *in vitro* conditions has not been systematically examined. The increasing demand for plant-derived bioactive compounds and concerns regarding the sustainability of wild plant harvesting have motivated the development of biotechnological approaches for the controlled production of secondary metabolites. Plant cell, tissue, and organ culture systems, including protocorm-like body (PLB) cultures, offer one such approach as they allow scalable year-round production of target compounds under defined, reproducible conditions (Georgiev et al., 2009). Among these systems, PLB-derived liquid cultures are particularly suitable for elicitation studies because they provide homogeneous, rapidly proliferating, and physiologically responsive tissue that permits uniform elicitor exposure and is readily amenable to scale-up. Only one study by Chusrisom et al. (2025) reported the production of bioactive compounds in *G. speciosum* using bioreactor cultures of PLB-derived materials under NaCl-mediated abiotic stress. Elicitation is one of the most effective strategies for enhancing secondary metabolite production in plant cells and organ cultures, as it mimics environmental or biotic stress signals that activate defense-related biosynthetic pathways. Methyl jasmonate (MeJA), a signaling molecule involved in the octadecanoid pathway, is one of the most widely used and effective chemical elicitors in plant biotechnology. MeJA induces phenylpropanoid and flavonoid biosynthesis by upregulating key biosynthetic enzymes, including PAL, 4CL, CHS, and CHI (Li et al., 2022; Zeng et al., 2025). Because PAL controls the entry of carbon into phenylpropanoid metabolism, MeJA-induced upregulation of PAL is expected to channel additional substrates toward the flavonoid branch and ultimately toward C-glycosylflavone end products, such as vitexin. The response to MeJA is typically dose-dependent and often biphasic, with moderate concentrations stimulating secondary metabolism, whereas excessive elicitor doses can cause stress and reduce metabolic efficiency (Ho et al., 2020). MeJA elicitation has been successfully applied to various plant cell and organ culture systems to enhance the production of specific bioactive compounds such as phenolics in *Echinacea purpurea* (Demirci, 2022), flavones in *Scutellaria baicalensis* (Geng et al., 2023), and alkaloids in *Catharanthus roseus* (Ruiz-May et al., 2011). Despite the well-established effectiveness of MeJA, its effects on *G. speciosum* PLB-derived liquid cultures, particularly vitexin accumulation and PAL activity, have not been investigated. This present study was designed to establish PLB-derived liquid cultures of *G. speciosum* and to evaluate the effects of MeJA at various concentrations on growth kinetics, total phenolic and flavonoid accumulation, vitexin production, PAL activity, antioxidant capacity, and enzyme inhibition.

## 2 Materials and methods

### 2.1 Plant material and induction of protocorm-like bodies

*G. speciosum* Blume was authenticated by the Queen Sirikit Botanic Garden Herbarium, Plant Varieties Protection Division, Department of Agriculture, Thailand, and a voucher specimen was deposited under the accession number BKs01704. Protocorm-like bodies (PLBs) were induced from shoot apices excised from aseptically grown *G. speciosum* plantlets. The explants were cultured on Vacin and Went (VW) medium (PhytoTechnology Laboratories, Shawnee Mission, KS, USA), a mineral formulation widely used for orchid seed germination and PLB induction (Vacin and Went, 1949), supplemented with 20 g/L sucrose, 1.0 mg/L 6-benzylaminopurine (BAP), and 0.5 mg/L 1-naphthaleneacetic acid (NAA). Approximately five explants, each 3–5 mm in length, were placed per 90 mm Petri dish and incubated under sterile conditions for PLB induction. The cultures were maintained at 25 ± 2 °C under a 16 h light/8 h dark photoperiod provided by cool-white, fluorescent lamps at a photon flux density of approximately 40 µmol m^-2^ s^-1^. PLBs are somatic-embryo-like structures that proliferate rapidly and uniformly, providing reproducible source material for liquid culture and elicitation studies. The induced PLBs were subcultured at 4-week intervals and subsequently used as the source material for establishing PLB-derived liquid cultures. An overview of the experimental workflow is presented in **Supplementary Figures S1** and **S2**.

### 2.2 Establishment and growth kinetics of PLB-derived liquid cultures

PLBs of *G. speciosum* were used as inocula to establish liquid cultures. Approximately 10 g fresh weight of PLBs was transferred to 150 mL of liquid VW medium (free of plant growth regulators) in a 250 mL culture flask and cultured on an orbital shaker (120 rpm) at 25 ± 2 °C under a 16 h light/8 h dark photoperiod for 40 days. To determine the growth kinetics, cultures were harvested at 4-day intervals (0, 4, 8, 12, 16, 20, 24, 28, 32, 36, and 40). At each sampling point, the biomass was separated from the culture medium by filtration through Whatman No. 1 filter paper, dried at 50 °C for 8 h, and weighed. The dry growth index (DGI) was calculated as follows:


$$\text{DGI}=({\text{W}}_{\text{t}}-{\text{W}}_{0})/{\text{W}}_{0}$$

where W_0_ is the initial dry biomass of the culture, and W_t_ is the dry biomass at the indicated cultivation time.

### 2.3 Methyl jasmonate elicitation

PLB-derived liquid cultures were established as described in 2.2. Approximately 20 g fresh weight of 4-week-old PLBs was inoculated into 250 mL of liquid VW medium in 500 mL culture flasks and maintained at 25 ± 2 °C under a 16 h light/8 h dark photoperiod with continuous agitation at 120 rpm. A MeJA stock solution was prepared in 95% ethanol and filter-sterilized through a 0.22 µm membrane before aseptic addition to the cultures. After 7 days of culture, MeJA was added at final concentrations of 0, 50, 75, and 100 µM. The control treatment received an equal volume of 95% ethanol (final concentration ≤ 0.05% v/v) without MeJA. Each treatment was performed in triplicate (three independent flasks per treatment). Biomass samples for biochemical analyses were collected on Days 0, 3, 6, 9, and 12 post-elicitation. The elicitation period was limited to 12 days to capture MeJA-induced secondary metabolite biosynthesis while minimizing potential growth inhibition and biomass reduction caused by prolonged elicitor exposure. This timeframe is consistent with previous elicitation optimization studies in plant cell cultures (Wang et al., 2016; Nhan and Loc, 2018).

### 2.4 Preparation of cell extracts

The harvested biomass was freeze-dried and ground into fine powder. Extraction was performed as described by Ali and Abbasi (2014), with minor modifications. Briefly, 100 mg of freeze-dried biomass was extracted with 10 mL of absolute ethanol containing 0.2% (v/v) acetic acid by ultrasonication for 10 min. Acidified ethanol extracts phenolics and flavonoids, limits oxidative degradation during ultrasonication, and matches the HPLC mobile phase (Chaves et al., 2020; Zhong et al., 2019). The mixture was incubated at room temperature for 30 min and centrifuged at 8,000 rpm for 10 min. The supernatant was collected, yielding an extract equivalent to 10 mg dry biomass per mL and stored at −20 °C until further analysis. The extracts were used to determine total phenolic and flavonoid contents, vitexin content, antioxidant activities, and enzyme inhibition.

### 2.5 Determination of total phenolic content

The total phenolic content (TPC) was determined using the Folin-Ciocalteu colorimetric method adapted from Slinkard and Singleton (1977), with slight modifications. Briefly, 20 µL of *G. speciosum* extract was added to a 96-well microplate and mixed with 100 µL of 10% (v/v) Folin-Ciocalteu reagent. The mixture was then incubated at room temperature for 7 min. Thereafter, 80 µL of 7.5% (w/v) sodium carbonate solution was added, followed by incubation at room temperature for 60 min. The absorbance was measured at 760 nm using a microplate reader (Infinite M Nano; Tecan, Männedorf, Switzerland). Gallic acid (Sigma-Aldrich, St. Louis, MO, USA) was used to prepare a standard calibration curve, and the results were expressed as milligrams of gallic acid equivalents per gram of culture dry weight (mg GAE/g CDW).

### 2.6 Determination of total flavonoid content

Total flavonoid content (TFC) was quantified using the aluminum chloride colorimetric method adapted from Ghasemzadeh et al. (2015) with slight modifications. Briefly, 30 µL of extract was added to a 96-well microplate, followed by 100 µL of NaNO_2_ solution (1:5, w/v). After incubation at room temperature for 6 min, 15 µL of AlCl_3_ solution (1:10, w/v) was added, and the mixture was allowed to stand for another 6 min. Subsequently, 55 µL of 1 M NaOH was added, and the reaction mixture was incubated at room temperature for 10 min. Absorbance was measured at 510 nm using a microplate reader. Quercetin (Sigma-Aldrich) was used as the standard, and the results were expressed as milligrams of quercetin equivalents per gram of culture dry weight (mg QE/g CDW).

### 2.7 HPLC-DAD analysis of vitexin

The vitexin content was determined using high-performance liquid chromatography with a diode array detector (HPLC-DAD) according to Zhang et al. (2022), with slight modifications. The analysis was performed using a Shimadzu HPLC system equipped with an SPD-M20A diode array detector (Shimadzu, Kyoto, Japan). Separation was carried out on an RP-HPLC ODS-3 C18 column (4.6 × 150 mm, 5 µm) at 35 °C with a flow rate of 1.0 mL min^-1^ and an injection volume of 5 µL; detection was performed at 336 nm. The mobile phase consisted of acetonitrile (A) and 0.2% (v/v) acetic acid in water (B). The gradient elution program was as follows: 0–30 min, 5–35% A; 30–31 min, 35–5% A; and 31–40 min, 5% A, for column re-equilibration. A standard stock solution of vitexin (0.1 mg mL^-1^) was prepared in methanol, and calibration was performed over the range 1–100 µg mL^-1^ with good linearity (*R*^2^ = 0.9971). Vitexin in the samples was identified by comparing the retention time and UV absorption spectrum with the authentic standard, and quantification was performed using an external calibration curve. Each sample was analyzed in triplicate, and vitexin compound was expressed as µg vitexin/g CDW. In addition, vitexin yield per flask was calculated as vitexin content (µg/g CDW) × final dry biomass per flask (W_t_, g DW), where W_t_ is the mean dry biomass at Day 12.

### 2.8 Vitexin identification using LC-ESI-QTOF-MS

The vitexin peak quantified by HPLC-DAD was confirmed by LC-ESI-QTOF-MS using an Agilent 6545 Q-TOF system equipped with a Dual AJS ESI source (Agilent Technologies, Santa Clara, CA, USA). Separation was performed on a Poroshell EC-C18 column at 35 °C using 0.2% acetic acid in water (A) and 0.2% acetic acid in acetonitrile (B) at a flow rate of 0.2 mL/min. The gradient was 5% B for 0–1 min, 5–17% B for 1–10 min, 17% B for 10–13 min, 17–100% B for 13–20 min, 100% B for 20–25 min, 100-5% B for 25–27 min, and 5% B for 27–33 min. The injection volume was 1 µL. MS data were acquired in positive ESI mode over m/z 100-1100, with Auto-MS^2^ scanning from m/z 50-1100 (MS scan rate, 2 spectra/s; MS/MS scan rate, 3 spectra/s; isolation width, ~4 amu). The main source conditions were as follows: gas temperature 300 °C, drying gas flow 10 L/min, nebulizer pressure 35 psig, sheath gas temperature 250 °C, sheath gas flow 11 L/min, capillary voltage 3500 V, nozzle voltage 500 V, fragmentor voltage 135 V, skimmer voltage 65 V, and octopole RF peak 750 V. Data were processed using MassHunter software. Vitexin was identified by its retention behavior and accurate mass, showing [M+H]^+^ at m/z 433.1132 and [M+Na]^+^ at m/z 455.0940, with mass errors below 2 ppm.

### 2.9 Phenylalanine ammonia-lyase (PAL) activity assay

PAL (EC 4.3.1.24) activity was determined according to the method described by Liu et al. (2019), with minor modifications. Fresh cells (2 g) were ground in liquid nitrogen and homogenized in 10 mL of ice-cold 50 mM sodium borate buffer (pH 8.8) containing 5 mM β-mercaptoethanol, 2 mM EDTA, and 40 g L^-1^ polyvinylpyrrolidone. After centrifugation at 13,000 × *g* for 20 min at 4 °C, the supernatant was used as the crude enzyme extract. The assay mixture contained 0.1 mL crude enzyme extract, 2 mM L-phenylalanine, and 20 mM sodium borate buffer (pH 8.8). After incubation at 30 °C for 1 h, PAL activity was determined from the increase in absorbance at 290 nm due to trans-cinnamic acid formation. One unit of PAL activity was defined as an increase in absorbance of 0.01 at 290 nm per hour, and the activity was expressed as U g^-1^ fresh cell weight (U g^-1^ FCW).

### 2.10 Antioxidant activity assays

#### 2.10.1 DPPH radical scavenging activity

DPPH radical scavenging activity was determined using an assay adapted from Rungruang et al. (2021) with slight modifications. Briefly, 100 µL of *G. speciosum* extract was mixed with 100 µL of 0.1 mM DPPH solution in ethanol in a 96-well microplate. The reaction mixture was incubated in the dark at room temperature for 30 min, and the absorbance was measured at 517 nm using a microplate reader. Trolox (Sigma-Aldrich) was used as the standard, and antioxidant activity was expressed as milligrams of Trolox equivalents per gram of culture dry weight (mg TE/g CDW).

#### 2.10.2 ABTS radical cation scavenging activity

The ABTS radical scavenging activity was measured according to the method described by Cai et al. (2004), with slight modifications. The ABTS stock solution was prepared by mixing 7 mM ABTS with 2.45 mM potassium persulfate at 2:1 (v/v), followed by incubation in the dark at room temperature for 12–16 h to allow radical formation. Before analysis, the stock solution was diluted with 95% ethanol to obtain an absorbance of 0.700 ± 0.030 at 734 nm. For the assay, 40 µL of extract was mixed with 160 µL of diluted ABTS^•+^ solution in a 96-well microplate and incubated at room temperature for 6 min. The absorbance was measured at 734 nm. Trolox (Sigma-Aldrich) was used as the standard, and the results were expressed as mg TE/g CDW.

#### 2.10.3 Copper-chelating activity

Copper-chelating activity was determined according to the method described by Rungruang et al. (2021) with slight modifications. Two hundred and ninety microliters of 50 mM sodium acetate buffer (pH 6.0) was mixed with 10 µL of extract; 6 µL of 2 mM pyrocatechol violet was then added, followed by 10 µL of 100 mg L^-1^ copper(II) sulfate. After incubation for 2 min at room temperature, the absorbance was measured at 632 nm using a microplate reader. EDTA (Sigma-Aldrich) was used as the standard, and the results were expressed as milligrams of EDTA equivalents per gram of culture dry weight (mg EDTA/g CDW).

### 2.11 Enzyme inhibition assay

#### 2.11.1 Anti-collagenase activity assay

Collagenase inhibitory activity was determined using the EnzChek Gelatinase/Collagenase Assay Kit (E12055, Invitrogen, Thermo Fisher Scientific), with minor modifications adapted from Rungruang et al. (2021). The sample reaction (200 µL) contained 80 µL of diluted extract (2.5 µL extract in 1× reaction buffer; final 125 µg dry biomass equivalent/mL), 100 µL of collagenase from *Clostridium histolyticum*, and 20 µL of DQ collagen type I substrate in a 96-well black microplate. Corresponding positive control (enzyme without extract), negative control (without enzyme), and sample blank (extract and substrate without enzyme) were run in parallel; each adjusted to 200 µL with 1× reaction buffer. After incubation in the dark at 25 °C for 90 min, fluorescence was measured at excitation/emission wavelengths of 485/538 nm. The inhibitory activity was expressed as the percentage of inhibition relative to the solvent control.

#### 2.11.2 Anti-elastase activity assay

Elastase inhibitory activity was determined by measuring the release of *p*-nitroaniline from N-succinyl-Ala-Ala-Ala-*p*-nitroanilide, as described by Rungruang et al. (2021) and Kim et al. (2010), with minor modifications. Porcine pancreatic elastase and its substrates were purchased from Sigma-Aldrich. The sample reaction (200 µL) contained 20 µL of diluted extract (2.5 µL extract in 0.1 M Tris-HCl buffer, pH 8.0; final 125 µg dry biomass equivalent/mL) and 160 µL of N-succinyl-Ala-Ala-Ala-*p*-nitroanilide (final 0.88 mM). After pre-incubation at 25 °C for 5 min, the reaction was initiated by adding 20 µL of porcine pancreatic elastase (final 0.0025 U/mL) and incubated at 25 °C for 30 min. Absorbance was measured at 410 nm using a microplate reader (Infinite 200 Pro, Tecan, Switzerland), with appropriate enzyme and substrate controls run in parallel. The elastase inhibitory activity was expressed as the percentage of inhibition relative to the solvent control.

#### 2.11.3 Anti-tyrosinase activity assay

Tyrosinase inhibitory activity was determined using an L-DOPA oxidation assay adapted from Rungruang et al. (2021). The sample reaction (200 µL) contained 20 µL of diluted extract (5 µL extract in 50 mM sodium phosphate buffer, pH 6.8; final 250 µg dry biomass equivalent/mL), 140 µL of the same buffer, 20 µL of tyrosinase (final 10 units/mL), and 20 µL of 2.5 mM L-DOPA (final 0.25 mM) in a 96-well microplate. After incubation at 37 °C, absorbance was measured at 475 nm using a microplate reader, with the appropriate enzyme and substrate controls run in parallel. The tyrosinase inhibitory activity was expressed as the percentage of inhibition relative to the solvent control.

### 2.12 Statistical analysis

All measurements were performed in technical triplicate for each of three independent culture flasks (biological replicates, *n* = 3), and the technical replicates were averaged before statistical analysis. Data are presented as means ± SD. Normality and homogeneity of variance were verified by Shapiro-Wilk and Levene’s tests, respectively. The effects of MeJA concentration, cultivation day (or time, for PAL activity), and their interaction were assessed by two-way ANOVA, with full ANOVA outputs reported in **Supplementary Table S3**. Because the MeJA × cultivation day (or time) interaction was significant for all response variables, simple main effects were analyzed by one-way ANOVA, followed by Tukey’s HSD *post-hoc* comparisons among MeJA concentrations within each cultivation day; comparisons among cultivation days within the 75 µM MeJA treatment, indicated by asterisks (*), were performed separately using one-way ANOVA followed by Tukey’s HSD test. Vitexin content was quantified only on Day 12 and was therefore analyzed by one-way ANOVA. Yield uncertainties were propagated from the independent SDs of vitexin content and dry biomass by combining relative errors in quadrature. Differences were considered statistically significant at *p* < 0.05. All statistical analyses were performed using SPSS version 28 (IBM Corp., Armonk, NY, USA; RRID: SCR_016479).

## 3 Results

### 3.1 Growth kinetics of *G. speciosum* PLB-derived liquid cultures

PLB-derived liquid cultures of *G. speciosum* showed gradual biomass accumulation over the 40-day cultivation period (**Figures 1** and **2**). Morphological observations in **Figure 1** showed that the cultures formed compact PLB clusters with a pale green to yellowish coloration during the early cultivation stages (Days 0–12). As cultivation progressed, the aggregates increased in size and became more friable and heterogeneous in appearance, particularly between Days 20 and 32. At later stages (Days 36–40), some aggregates appeared enlarged and translucent, and slight browning was observed in localized regions. The dry growth index (DGI) increased continuously from Days 0 to 32, reaching its highest value at Day 32 (0.72 ± 0.09); thereafter, a slight decline in DGI was observed at Days 36 and 40 (**Figure 2**). Therefore, the growth profile showed a gradual increase in biomass during the first 24 days, followed by a more rapid increase between Days 24 and 32, before the culture entered the stationary phase after Day 32.

**Figure 1:**
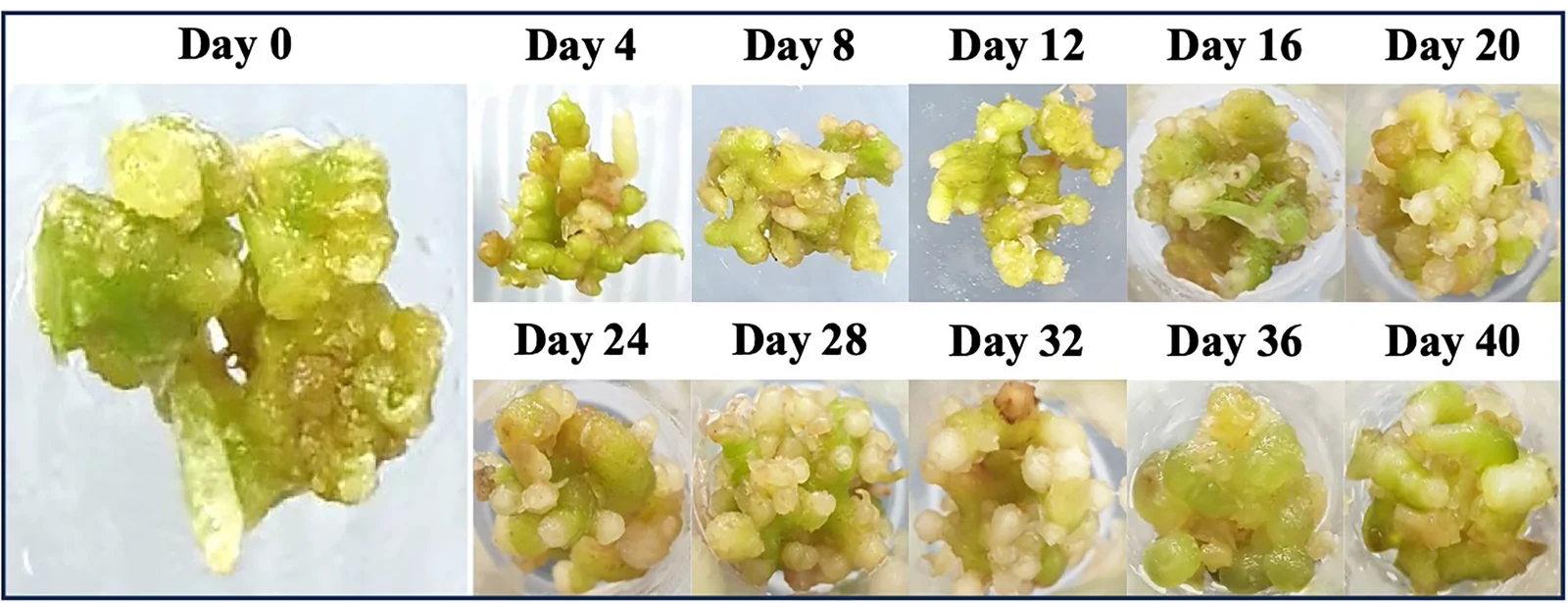
Morphological appearance of *G. speciosum* protocorm-like bodies (PLBs) cultured during 40 days of cultivation. Representative images of PLB clusters recorded at 4-day intervals from Days 0–40.

**Figure 2:**
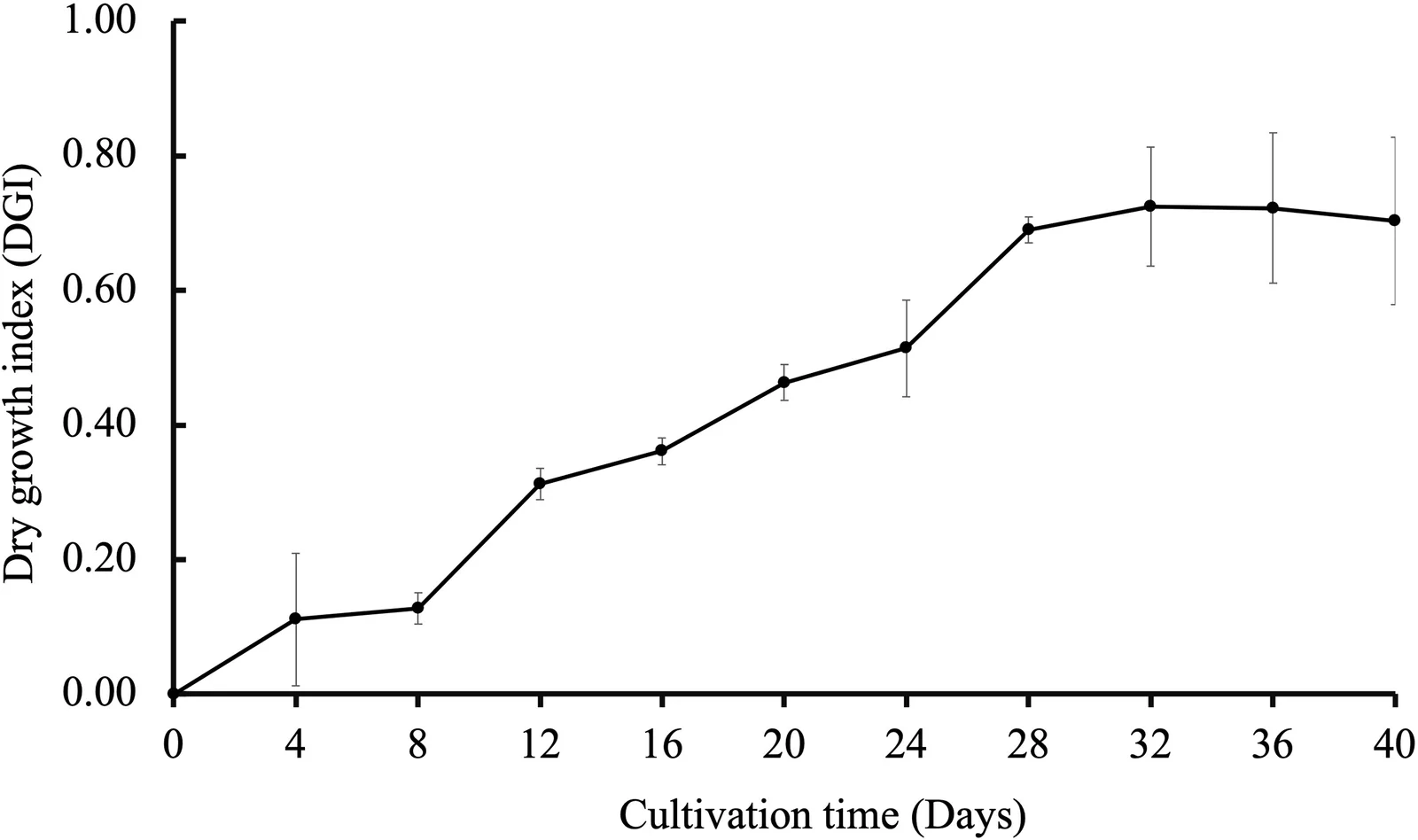
Growth kinetics of *G. speciosum* PLB-derived liquid cultures during 40 days of cultivation, expressed as dry growth index (DGI). Data are presented as means ± SD (*n* = 3).

### 3.2 Effects of MeJA on morphology and growth

The morphological appearance of *G. speciosum* PLB-derived liquid cultures after MeJA treatment is shown in **Figure 3**. The PLB clusters remained morphologically intact throughout the 12-day cultivation period in all treatment groups, although changes in PLB cluster size and coloration were observed at higher MeJA concentrations (**Figure 3**). Cultures treated with 75 and 100 µM MeJA gradually developed a more compact and yellowish appearance during prolonged cultivation compared with the untreated control. The effects of MeJA on plant growth are shown in **Figure 4**. In all treatments, the DGI increased progressively after elicitation; however, the untreated control showed the highest DGI throughout the experiment, reaching 0.089 ± 0.002 at Day 12. MeJA treatment reduced the DGI, with the strongest reduction observed at 100 µM. At Day 12, cultures treated with 50, 75, and 100 µM MeJA showed DGI values of 0.066 ± 0.007, 0.063 ± 0.002, and 0.050 ± 0.001, respectively (**Figure 4**). Despite this reduction in growth, the PLB aggregates remained morphologically intact throughout the experimental period.

**Figure 3:**
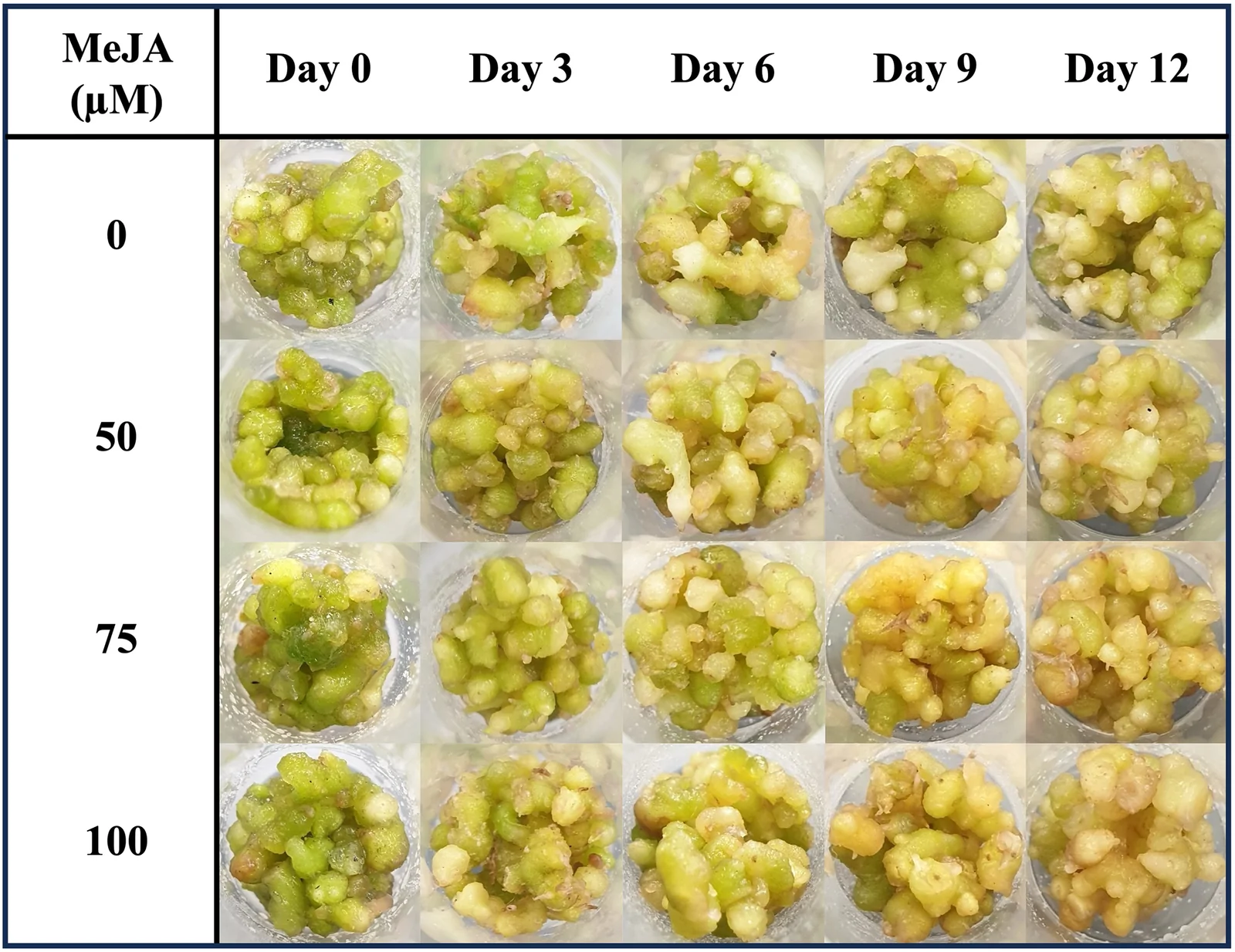
Morphological appearance of *G. speciosum* PLB-derived liquid cultures during MeJA elicitation. Cultures were pre-cultivated for 7 days then treated with MeJA at 0 (control), 50, 75, and 100 µM; the day of MeJA addition was designated as Day 0. Rows correspond to the four MeJA concentrations, and columns correspond to sampling times after elicitation (Days 0, 3, 6, 9, and 12). Each panel shows a representative culture selected from three independent biological replicates (n = 3).

**Figure 4:**
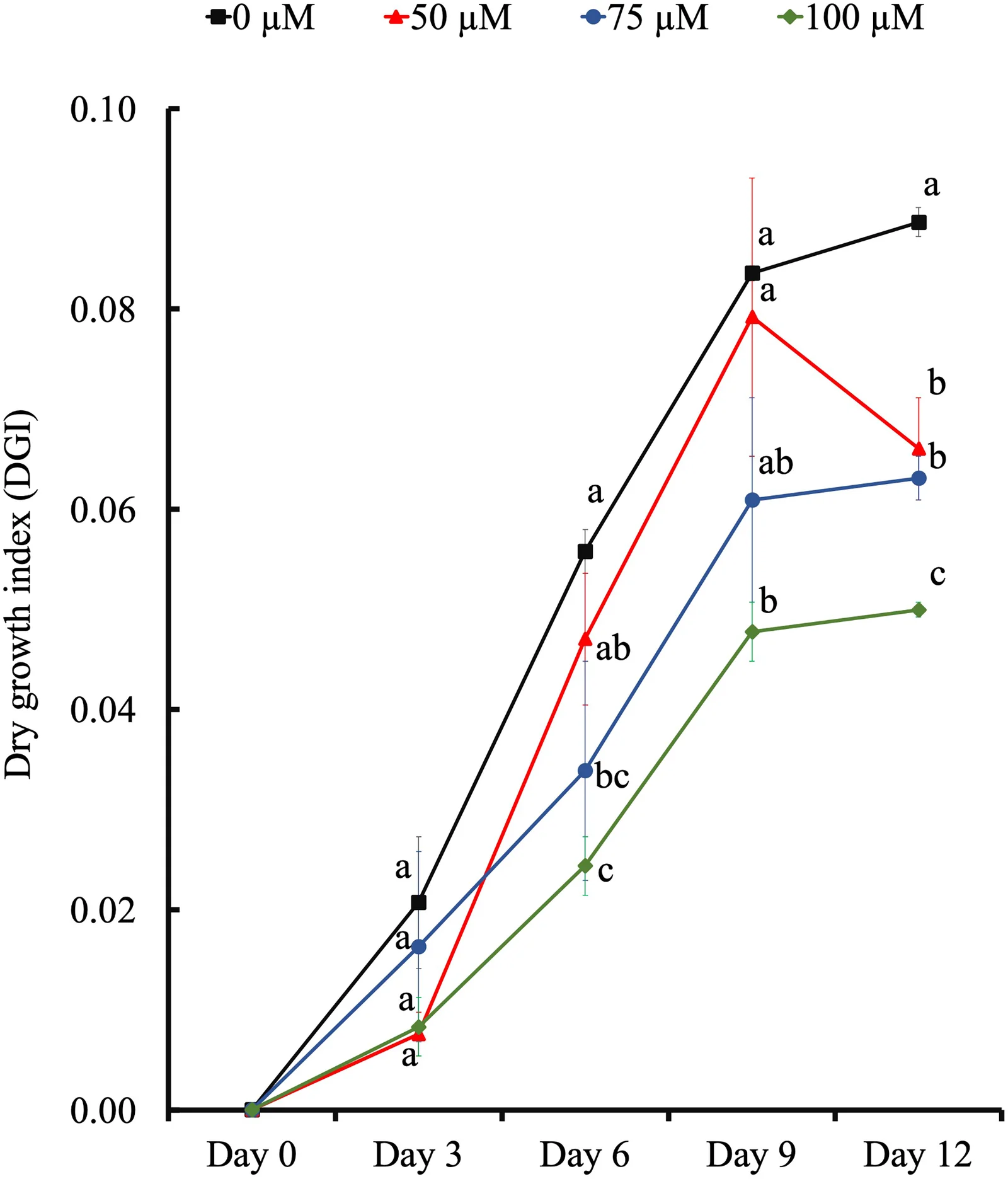
Effects of MeJA on the DGI of *G. speciosum* PLB-derived liquid cultures during the 12 days after elicitation. Data are presented as means ± SD (*n* = 3). Different lowercase letters indicate significant differences among MeJA concentrations within the same cultivation day (*p* < 0.05, Tukey’s HSD); for this figure, letters are assigned in descending order of DGI (a = highest). Full two-way ANOVA results are reported in **Supplementary Table S3**.

### 3.3 Effects of MeJA on total phenolic content (TPC) and total flavonoid content (TFC)

The effects of MeJA on the TPC and TFC are shown in **Figures 5** and **6**, respectively. Both TPC and TFC increased progressively during the cultivation period in all treatment groups; however, MeJA-treated cultures accumulated higher levels of phenolic and flavonoid compounds than the untreated controls. The TPC of the untreated control increased gradually from 3.340 ± 0.176 to 5.674 ± 0.278 mg GAE/g CDW at Days 0 and 12 (**Figure 5**). MeJA treatment enhanced TPC accumulation, particularly at 75 µM, which produced the highest value of 14.267 ± 0.273 mg GAE/g CDW at Day 12; the 50 µM and 100 µM treatments reached 11.316 ± 0.371 and 11.004 ± 0.319 mg GAE/g CDW, respectively. A similar pattern was observed for the TFC (**Figure 6**). The untreated control showed only a slight increase in flavonoid content from 3.080 ± 0.971 to 8.377 ± 0.747 mg QE/g CDW at Days 0 and 12, whereas MeJA treatment increased flavonoid accumulation substantially. Among all treatments, 75 µM MeJA produced the highest TFC value of 27.093 ± 0.504 mg QE/g CDW at Day 12, which was higher than that of the untreated control. Although 50 µM and 100 µM MeJA also enhanced TFC, the accumulation of TFC remained lower than that at 75 µM MeJA elicitation, particularly at Day 12.

**Figure 5:**
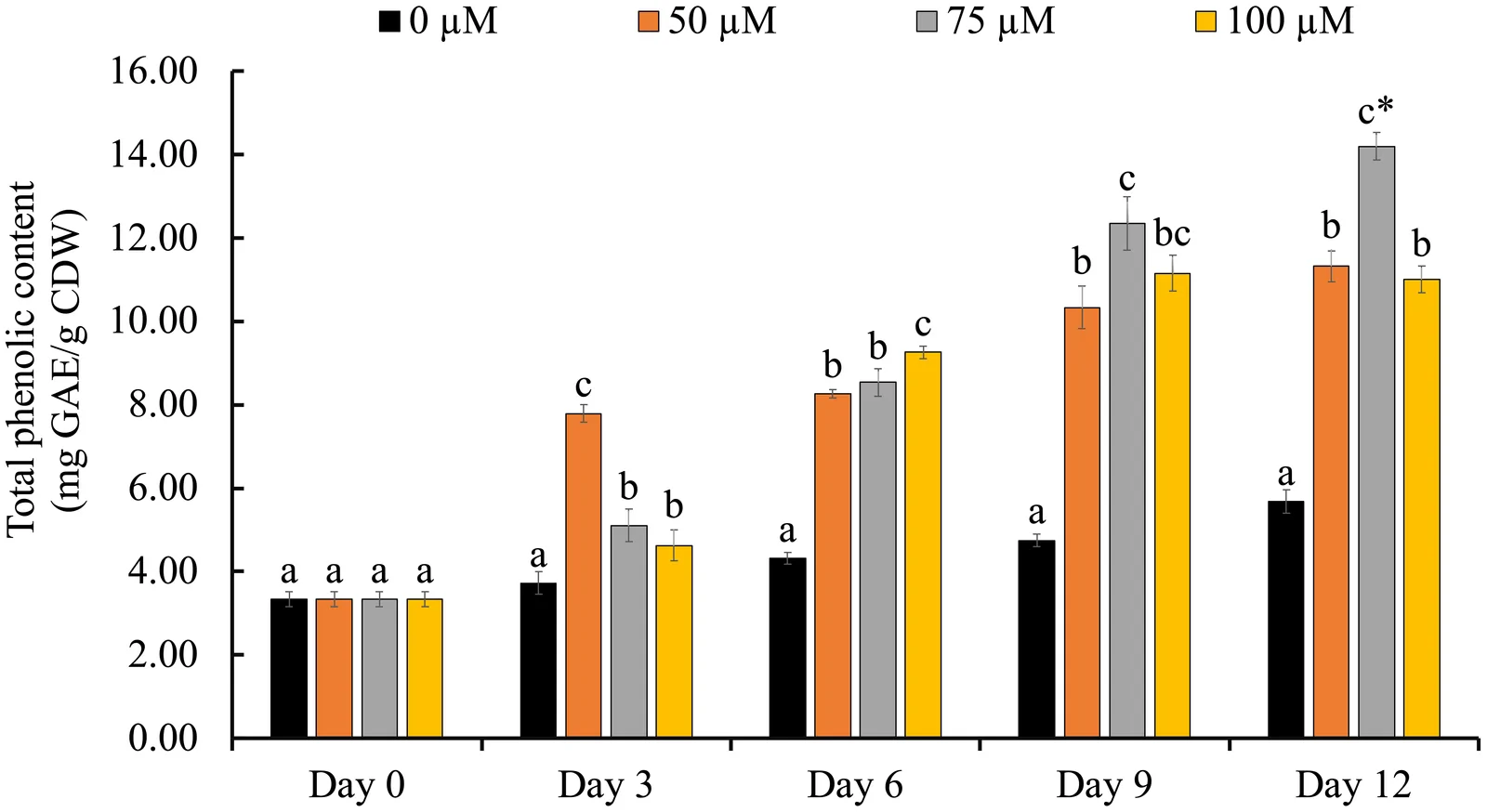
Effects of MeJA on TPC of *G. speciosum* PLB-derived liquid cultures during a 12 day cultivation period. Data are presented as means ± SD (*n* = 3). Different lowercase letters indicate significant differences among MeJA concentrations within the same cultivation day (*p* < 0.05, Tukey’s HSD). The asterisk (*) at Day 12 for the 75 µM MeJA treatment indicates the highest value among cultivation days within the same treatment (*p* < 0.05, Tukey’s HSD). Full two-way ANOVA results are reported in **Supplementary Table S3**.

**Figure 6:**
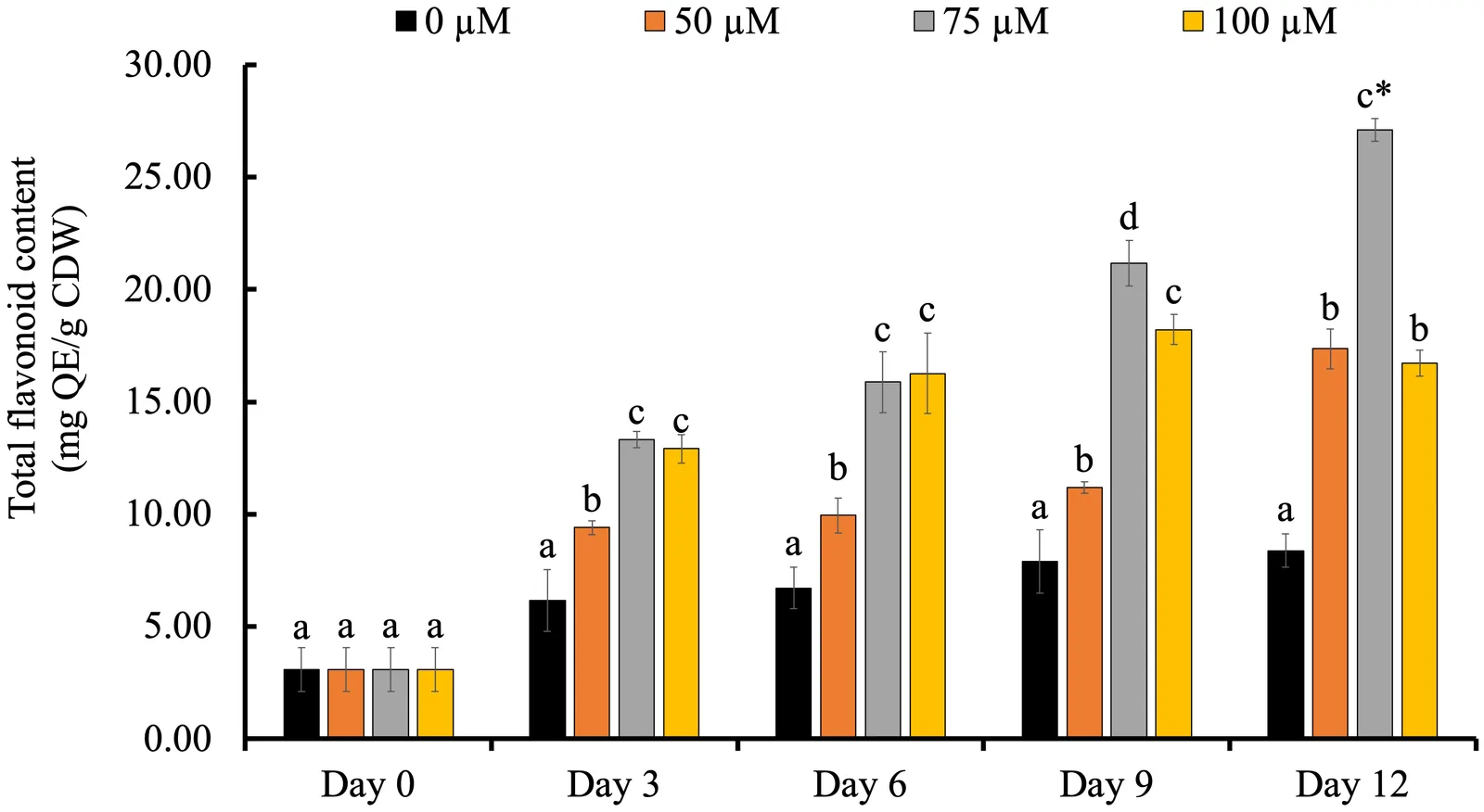
Effects of MeJA on TFC of *G. speciosum* PLB-derived liquid cultures during a 12 day cultivation period. Data are presented as means ± SD (*n* = 3). Different lowercase letters indicate significant differences among MeJA concentrations within the same cultivation day (*p* < 0.05, Tukey’s HSD). The asterisk (*) at Day 12 for the 75 µM MeJA treatment indicates the highest value among cultivation days within the same treatment (*p* < 0.05, Tukey’s HSD). Full two-way ANOVA results are reported in **Supplementary Table S3**.

### 3.4 Identification and quantification of vitexin by HPLC-DAD

Vitexin was identified in *G. speciosum* PLB-derived liquid culture extracts by comparing its retention time and UV absorption spectrum with those of an authentic standard (**Figure 7**; **Supplementary Table S1**). Quantification was performed using an external standard calibration curve, which showed good linearity (*R*^2^ = 0.9971) at 1–100 µg/mL. On Day 12, the vitexin content differed significantly among all treatments (*p* < 0.05). The untreated control contained 208.33 ± 2.69 µg/g CDW, whereas cultures treated with 50, 75, and 100 µM MeJA contained 302.14 ± 1.98, 420.05 ± 9.96, and 326.22 ± 3.01 µg/g CDW, respectively. Vitexin content increased with MeJA concentration up to 75 µM and declined at 100 µM. The highest vitexin content was obtained with 75 µM MeJA, corresponding to an approximately 2.02-fold increase compared with the untreated control at the same cultivation time. To account for the concomitant reduction in biomass under MeJA elicitation, vitexin yield per flask was also calculated (**Supplementary Table S2**). The 75 µM MeJA treatment reached 30.45 ± 0.75 µg vitexin/flask, approximately 1.97-fold higher than the untreated control (15.49 ± 0.21 µg/flask), and remained the highest yield among all treatments; the 50 and 100 µM treatments produced 21.93 ± 0.27 and 23.42 ± 0.22 µg/flask, corresponding to 1.42- and 1.51-fold increases, respectively.

**Figure 7:**
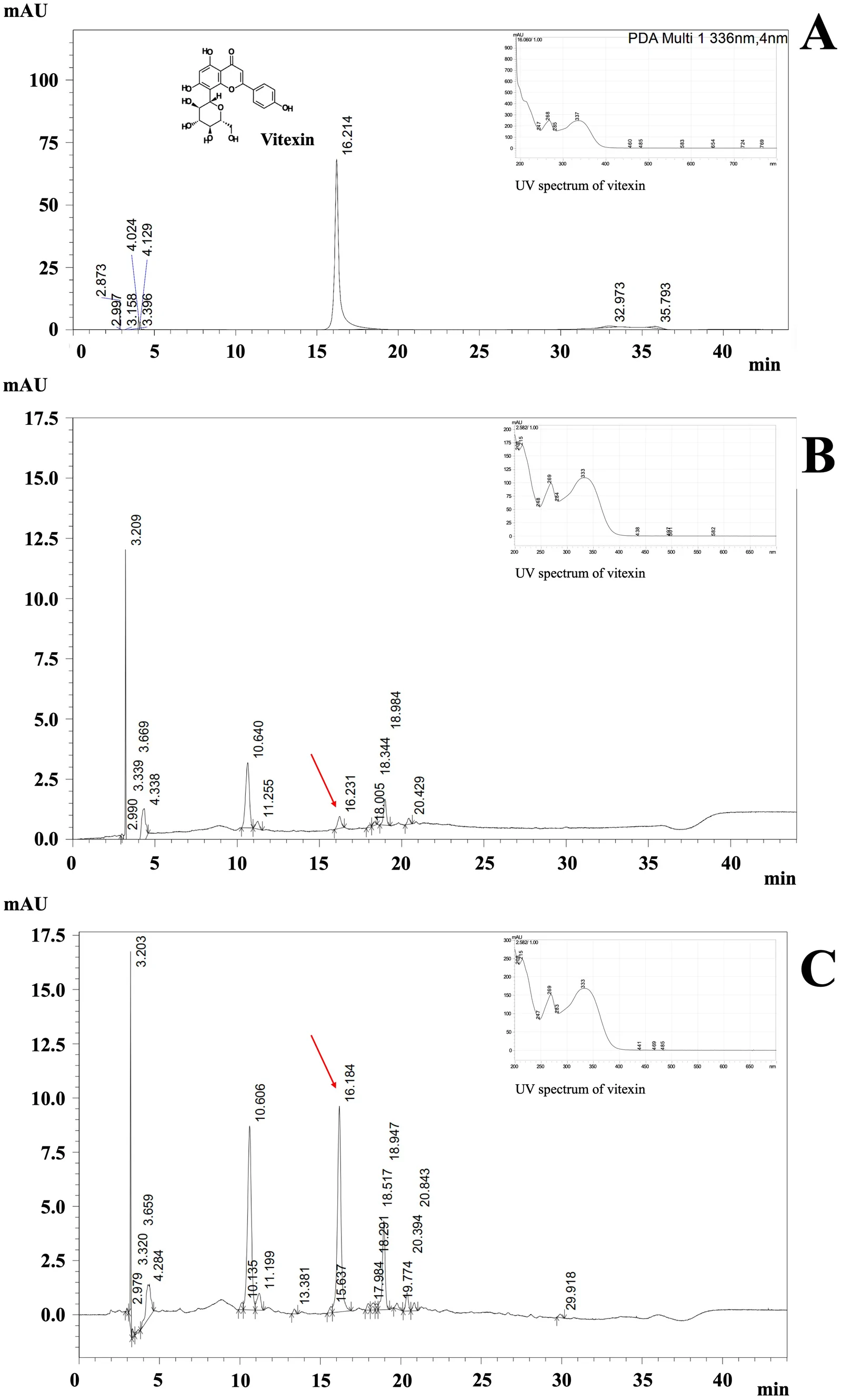
HPLC chromatograms and UV spectra of vitexin in *G. speciosum* PLB-derived liquid culture extracts after MeJA elicitation. **(A)** Vitexin standard, **(B)** control extract (0 µM MeJA, Day 12), and **(C)** MeJA-treated extract (75 µM MeJA, Day 12).

### 3.5 LC-ESI-QTOF-MS confirmation of vitexin identity

The vitexin peak detected by HPLC-DAD was confirmed by LC-ESI-QTOF-MS analysis. The chromatographic peak at retention time 15.31 min produced a mass spectrum dominated by a protonated molecular ion [M+H]^+^ at m/z 433.1132 and accompanied by a sodium adduct [M+Na]^+^ at m/z 455.0940 (**Figure 8**). The [M+H]^+^ mass deviated from the theoretical value for vitexin (C_21_H_20_O_10_; theoretical 433.1129) by -0.53 ppm, well within the tolerance accepted for Q-TOF identification (< 5 ppm). The isotope pattern (M + 1 at m/z 434.1164 and M + 2 at m/z 435.1185) and the sodium adduct mass error (+1.83 ppm) further matched the elemental composition of vitexin. Together with the HPLC retention time and UV-spectral match to the authentic standard, these data confirm the identity of the major C-glycosylflavone in MeJA-treated *G. speciosum* PLB-derived liquid cultures as vitexin (apigenin-8-C-β-D-glucopyranoside).

**Figure 8:**
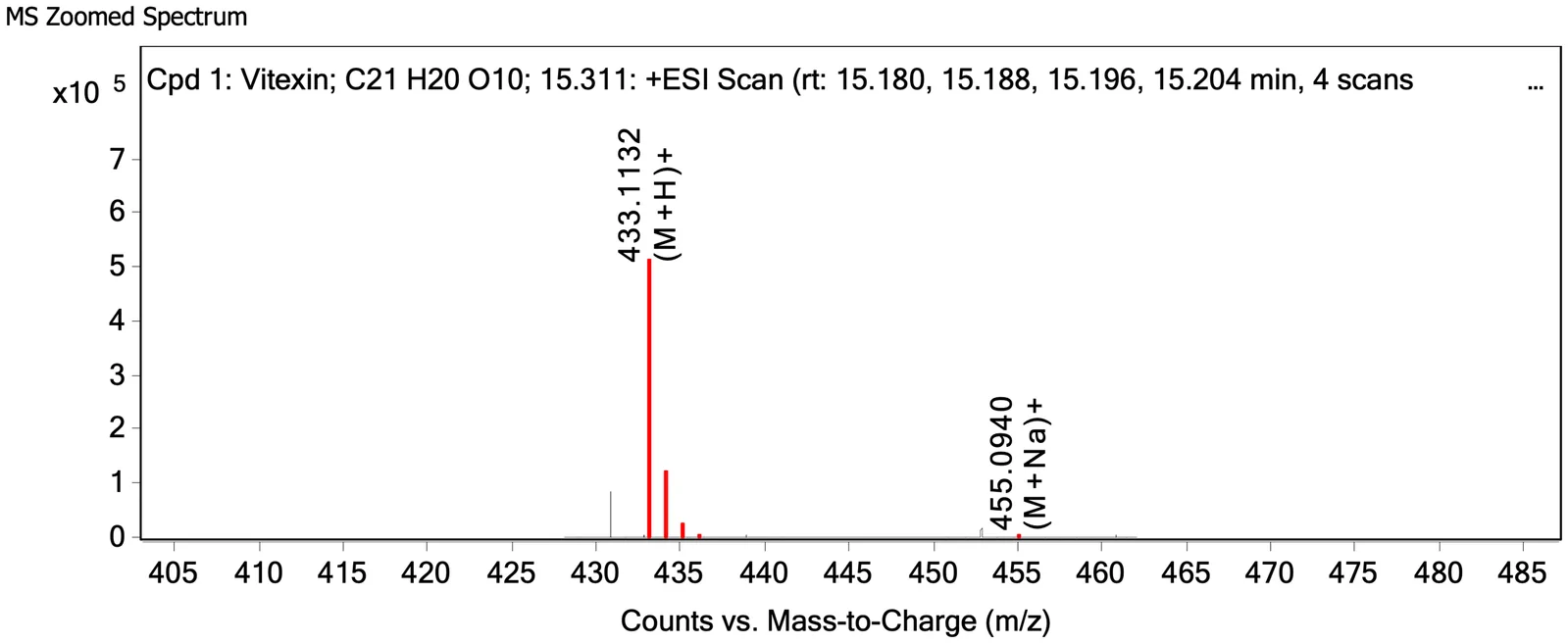
Confirmation of vitexin identity in *G. speciosum* PLB-derived liquid culture extracts by LC-ESI-QTOF-MS analysis. The +ESI scan spectrum averaged over four scans showed a protonated molecular ion [M+H]^+^ at m/z 433.1132 and a sodium adduct [M+Na]^+^ at m/z 455.0940.

### 3.6 Effects of MeJA on phenylalanine ammonia-lyase (PAL) activity

PAL, a key enzyme in phenylpropanoid metabolism, responded rapidly to MeJA elicitation in *G. speciosum* PLB-derived liquid culture (**Figure 9**). In the untreated control, PAL activity remained relatively stable throughout the 24 h experimental period, whereas all MeJA-treated cultures showed increased PAL activity within 6 h after elicitation. Among the elicited groups, 75 µM MeJA produced the highest activity, with the peak of 5.33 ± 0.54 U g^-1^ FCW recorded at 12 h. Cultures treated with 100 µM MeJA also showed a substantial increase in PAL activity, reaching 4.56 ± 0.22 U g^-1^ FCW at 18 h. In the 50 µM treatment, PAL activity increased rapidly during the first 12 h and declined thereafter. After 24 h, PAL activity declined in all MeJA-treated groups.

**Figure 9:**
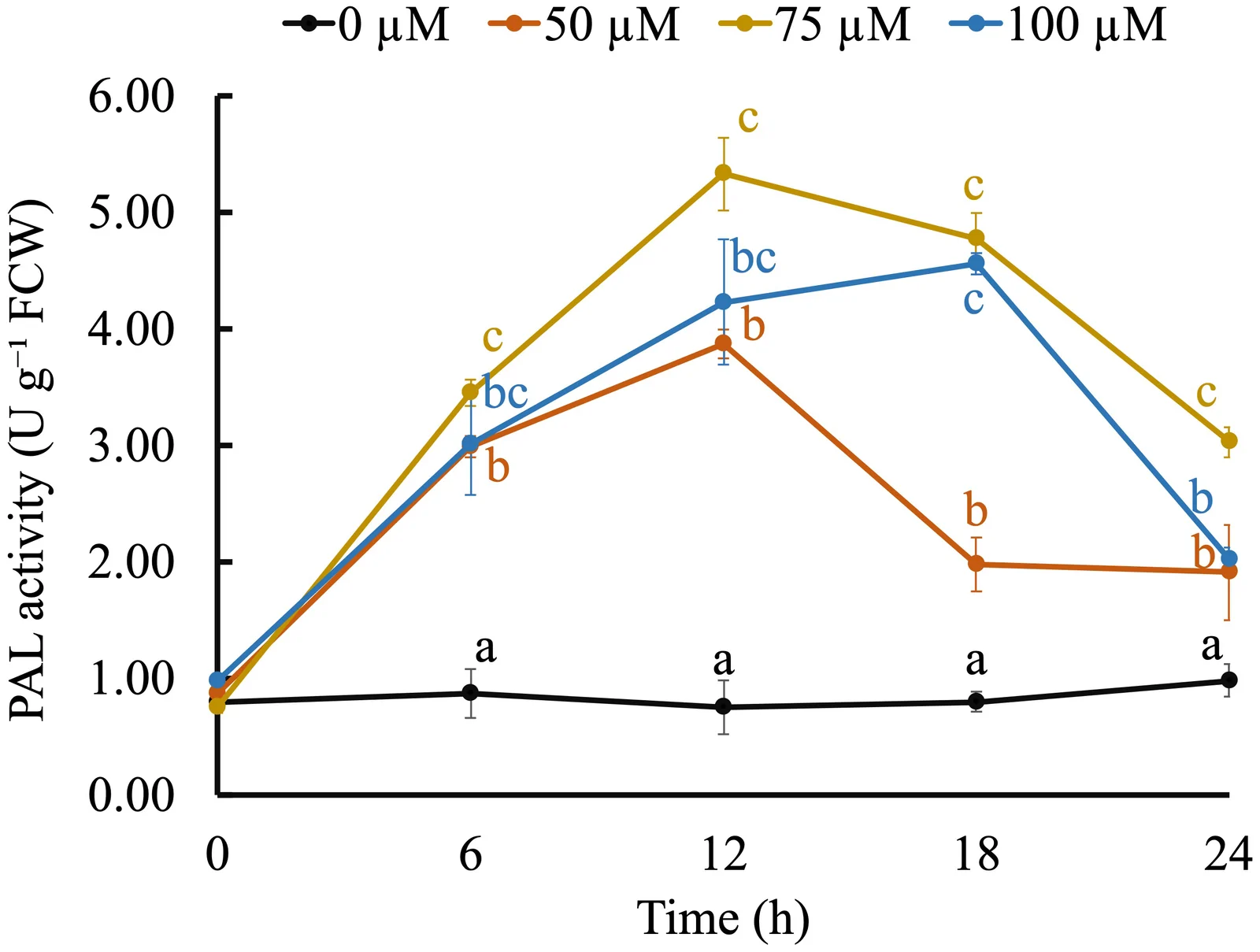
Effects of MeJA (0, 50, 75, and 100 µM) on PAL activity in *G. speciosum* PLB-derived liquid cultures during 24 h after elicitation. PAL activity was measured at 0, 6, 12, 18, and 24 h. Data are presented as means ± SD (*n* = 3). Different lowercase letters indicate significant differences among MeJA concentrations at the same sampling time (*p* < 0.05, Tukey’s HSD). Full two-way ANOVA results are reported in **Supplementary Table S3**.

### 3.7 Effects of MeJA on antioxidant activities

The DPPH radical scavenging capacity increased after MeJA treatment in all the treated culture extracts (**Figure 10A**). In control culture, DPPH radical scavenging activity increased from 1.09 ± 0.16 to 2.20 ± 0.11 mg TE/g CDW at Days 0 and 12, respectively. From Day 3 onward, MeJA-treated cultures showed higher DPPH scavenging capacity than the control, with the highest value observed at Day 12 with 75 µM MeJA (5.30 ± 0.20 mg TE/g CDW). The 50 µM and 100 µM MeJA treatments also enhanced DPPH scavenging capacity to 3.35 ± 0.05 and 3.75 ± 0.29 mg TE/g CDW, respectively; however, their activities remained lower than that of the 75 µM MeJA treatment at Day 12.

**Figure 10:**
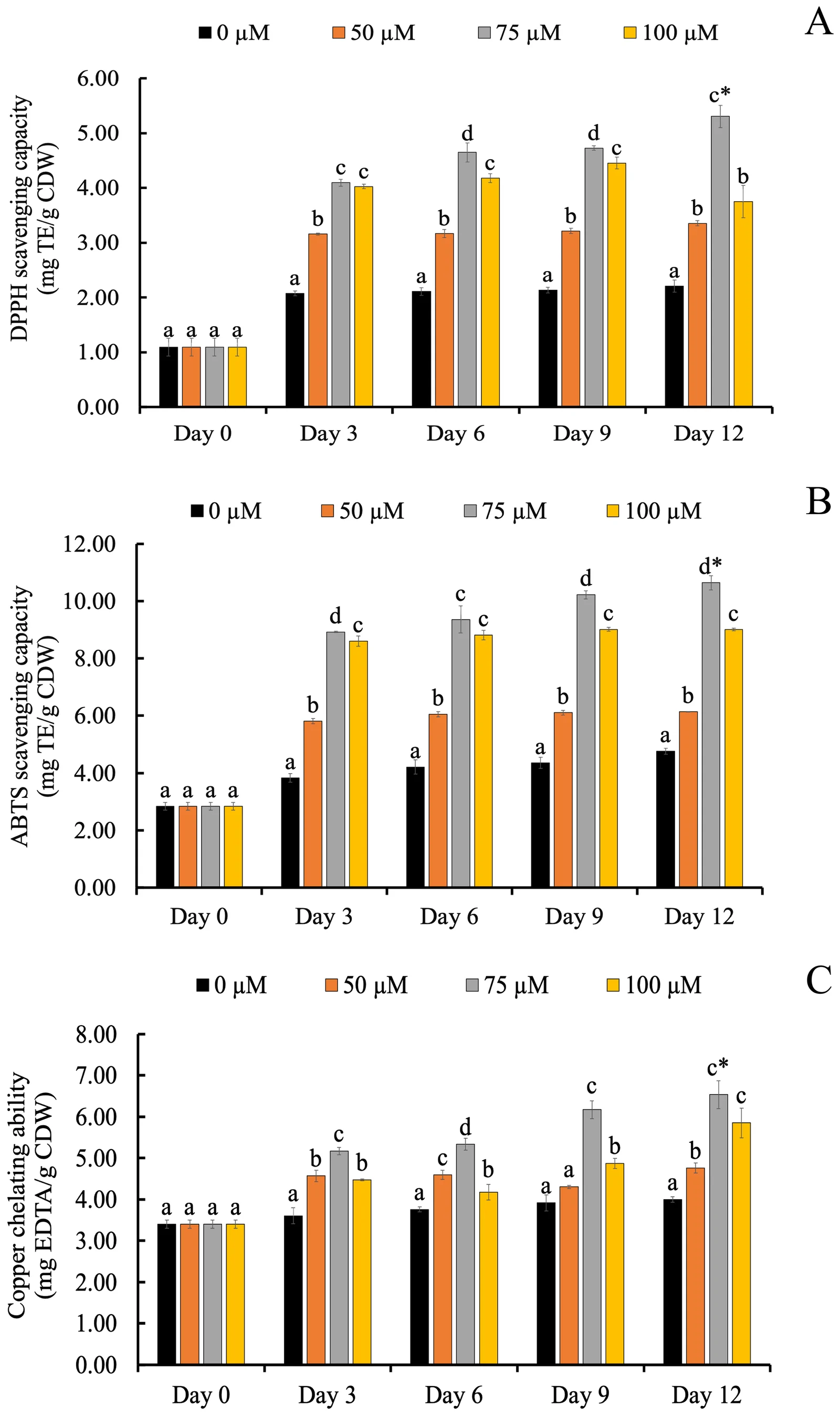
Effects of MeJA on antioxidant activities of *G. speciosum* PLB-derived liquid cultures during the 12 days after elicitation. **(A)** DPPH scavenging capacity, **(B)** ABTS scavenging capacity, and **(C)** copper chelating ability. Cultures were treated with MeJA at 0, 50, 75, and 100 µM. Data are presented as mean ± SD (*n* = 3). Different lowercase letters indicate significant differences among MeJA concentrations within the same cultivation day (*p* < 0.05, Tukey’s HSD). An asterisk (*) at Day 12 for the 75 µM MeJA treatment indicates the highest value among cultivation days within the same treatment for each assay (*p* < 0.05, Tukey’s HSD). Full two-way ANOVA results are reported in **Supplementary Table S3**.

The ABTS radical scavenging capacity showed a similar trend (**Figure 10B**). ABTS scavenging in the control cultures increased gradually from 2.84 ± 0.14 to 4.76 ± 0.10 mg TE/g CDW over 12 days, while MeJA treatment enhanced ABTS scavenging activity, with the highest activity again recorded at 75 µM (10.64 ± 0.25 mg TE/g CDW at Day 12); the 50 and 100 µM treatments reached 6.14 ± 0.01 and 9.01 ± 0.05 mg TE/g CDW, respectively.

Copper-chelating activity also increased during cultivation, particularly in the MeJA-treated cultures (**Figure 10C**). In the control cultures, copper-chelating activity increased slightly from 3.35 ± 0.12 mg EDTA/g CDW at Day 0 to 3.79 ± 0.35 mg EDTA/g CDW at Day 12. The highest copper-chelating activity was recorded in the 75 µM MeJA treatment at Day 12 (6.54 ± 0.29 mg EDTA/g CDW); although 50 and 100 µM MeJA improved copper-chelating activity to 4.82 ± 0.15 and 5.85 ± 0.31 mg EDTA/g CDW, respectively, these values remained lower than that of the 75 µM treatment.

### 3.8 Enzyme inhibitory activities

The effects of MeJA on the anti-collagenase, anti-elastase, and anti-tyrosinase activities of *G. speciosum* PLB-derived liquid culture extracts are shown in **Figure 11**.

**Figure 11:**
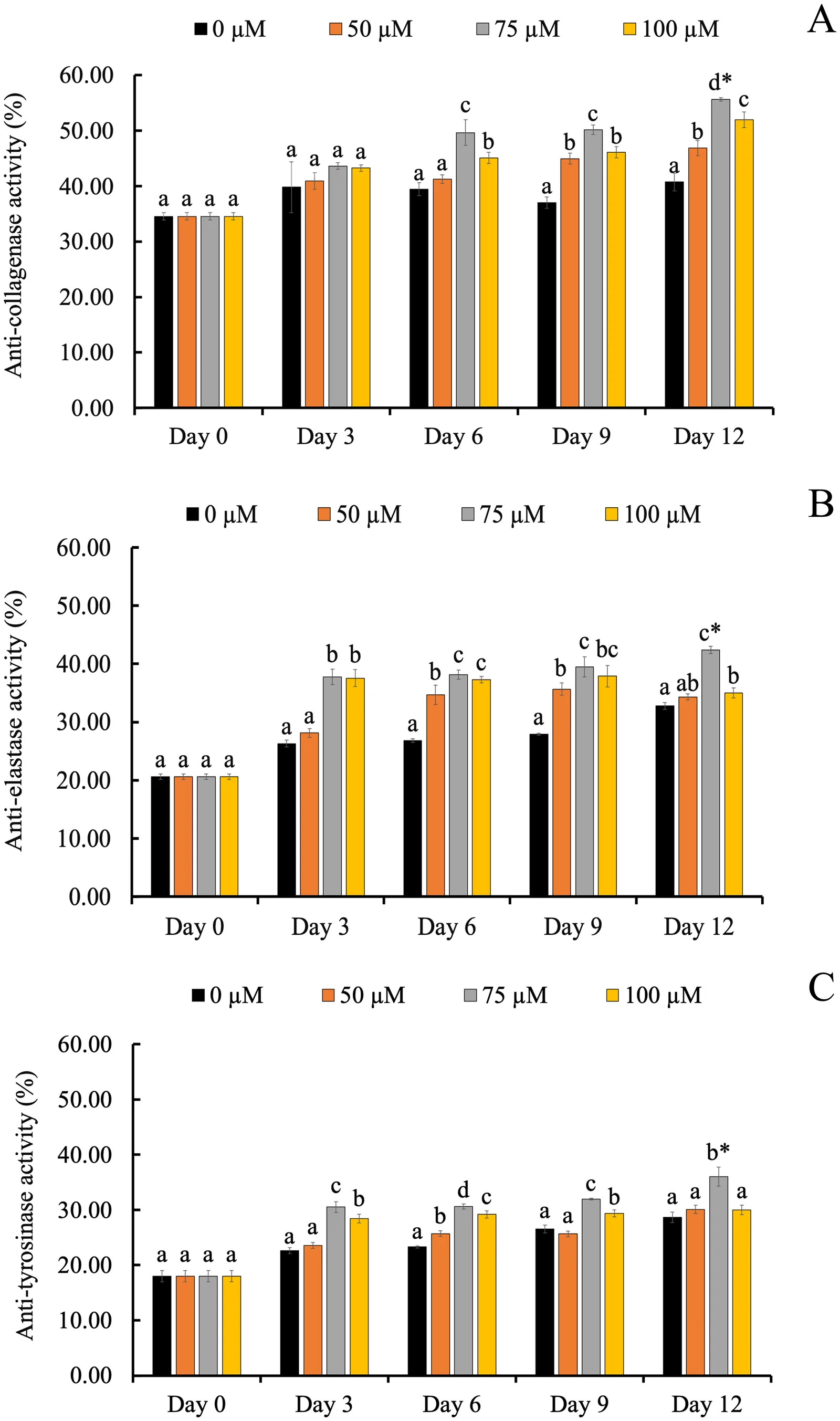
Effects of MeJA on anti-collagenase **(A)**, anti-elastase **(B)**, and anti-tyrosinase **(C)** activities of *G. speciosum* PLB-derived liquid cultures during the 12 days after elicitation. Cultures were treated with MeJA at 0, 50, 75, and 100 µM. Data are presented as mean ± SD (n = 3). Different lowercase letters indicate significant differences among MeJA concentrations within the same cultivation day (*p* < 0.05, Tukey’s HSD). An asterisk (*) at Day 12 for the 75 µM MeJA treatment indicates the highest value among cultivation days within the same treatment for each assay (*p* < 0.05, Tukey’s HSD). Full two-way ANOVA results are reported in **Supplementary Table S3**.

The anti-collagenase activity increased following MeJA elicitation throughout the cultivation period (**Figure 11A**). From Day 6 onward, cultures treated with 75 µM MeJA showed the highest anti-collagenase activity among all treatments, reaching 49.62 ± 2.32%, 50.15 ± 0.84%, and 55.62 ± 0.34% on Days 6, 9, and 12, respectively. At Day 12, the 75 µM MeJA activity (55.62 ± 0.34%) exceeded those of the 50 µM (46.84 ± 1.39%), 100 µM (51.95 ± 1.40%), and untreated control (40.73 ± 1.58%) treatments.

A similar increase was observed for anti-elastase activity (**Figure 11B**). On Day 3, cultures treated with 75 and 100 µM MeJA exhibited higher activities (37.74 ± 1.35% and 37.52 ± 1.46%, respectively) than the untreated control and 50 µM MeJA treatments. On Day 12, 75 µM MeJA produced the highest anti-elastase activity (42.40 ± 0.63%), exceeding the values of the cultures treated with 50 and 100 µM MeJA (34.32 ± 0.48 and 34.99 ± 0.85%, respectively) and the untreated control (32.77 ± 0.57%).

Anti-tyrosinase activity followed the same trend across the 12-day cultivation period (**Figure 11C**). From Days 6 to 12, cultures treated with 75 µM MeJA maintained the highest anti-tyrosinase activity, reaching 30.62 ± 0.48%, 31.94 ± 0.17%, and 36.01 ± 1.71% on Days 6, 9, and 12, respectively. At Day 12, the 75 µM MeJA activity (36.01 ± 1.71%) was higher than that of the 50 µM treatment (30.10 ± 0.71%), while the 100 µM (29.96 ± 0.86%) and the untreated control (28.63 ± 0.94%) values were similar to that of the 50 µM treatment.

## 4 Discussion

*G. speciosum* PLB-derived liquid cultures showed a typical batch-culture growth pattern with gradual biomass accumulation, followed by a plateau after Day 32. The sigmoidal profile reflects the progressive depletion of medium nutrients and the increasing mass-transfer constraints that characterize batch cultures of plant cells and organs (Georgiev et al., 2009). MeJA was applied on Day 7, while the cultures were still accumulating biomass and were therefore likely to retain the active primary metabolism needed to sustain elicitor-induced biosynthesis. Following MeJA treatment, biomass accumulation declined in a concentration-dependent manner, whereas the PLB clusters remained structurally intact. This response is consistent with a jasmonate-mediated growth-defense trade-off, in which primary growth is partially suppressed, whereas carbon flux is redirected toward defense-related secondary metabolism (Ho et al., 2020; Li et al., 2022). The yellowing and slight browning observed at higher MeJA concentrations were consistent with jasmonate-associated chlorophyll degradation and phenolic oxidation during stress-induced metabolic reprogramming (Zhu et al., 2017; Ho et al., 2020).

MeJA markedly enhanced TPC, TFC, and vitexin accumulation, with 75 µM producing the strongest response. The lower response at 100 µM suggests that excessive elicitor pressure imposed metabolic stress that limited biosynthetic efficiency, a common feature of MeJA elicitation in plant cell and organ cultures (Ho et al., 2020; Demirci, 2022; Woch et al., 2023). Physiologically, this biphasic dose–response may reflect the metabolic cost of elicitation. Above the optimal concentration, the oxidative stress generated by jasmonate signaling and the diversion of energy and reducing equivalents away from growth-related metabolism may constrain the resources available for phenylpropanoid biosynthesis (Ruiz-May et al., 2011). The concurrent decrease in biomass at 100 µM further suggests that the metabolic capacity supporting secondary-metabolite biosynthesis was compromised at this concentration. Notably, the enhancement differed across metabolite classes: at 75 µM MeJA on Day 12, TFC and TPC increased ~3.2- and 2.5-fold relative to the control, whereas vitexin increased only ~2-fold. Because metabolite contents are expressed per unit dry mass, the possibility that the observed increase in vitexin content was partly attributable to lower biomass yield was considered. However, yield calculations (**Supplementary Table S2**) showed that vitexin yield under 75 µM MeJA reached 30.45 ± 0.75 µg/flask, approximately 1.97-fold higher than the untreated control (15.49 ± 0.21 µg/flask), closely corresponding to the 2.02-fold increase in vitexin content. This indicates that the ~2.4% reduction in dry biomass had only a marginal effect on the overall increase in vitexin yield. The response therefore appears to reflect a genuine stimulation of biosynthesis rather than a passive concentration effect. We therefore hypothesize that MeJA stimulates the general phenylpropanoid/flavonoid flux more than the specific branch leading to vitexin. In this framework, the C-glycosylation step (C-glycosyltransferase) may act as a rate-limiting bottleneck that channels part of the additional flux toward other phenolics and co-occurring C-glycosylflavones rather than vitexin alone. The detection of vitexin in PLB-derived liquid cultures is particularly relevant because this C-glycosylflavone has previously been reported mainly in aerial tissues of *G. speciosum* (Yingchutrakul et al., 2021; Thamrongwatwongsa et al., 2024). Confirmation by HPLC-DAD and LC-ESI-QTOF-MS indicates that PLB-derived cultures of *G. speciosum* retain the biosynthetic capacity for C-glycosylflavone production, and that this capacity can be intensified by MeJA elicitation. However, earlier detections of vitexin in naturally grown *G. speciosum* were largely qualitative or based on untargeted metabolomics (Yingchutrakul et al., 2021), and a direct quantitative comparison between the culture-derived material (420.05 ± 9.96 µg g^-1^ CDW under 75 µM MeJA) and naturally grown tissues (leaf, pseudobulb, and flower) is the subject of a separate, ongoing study that will be reported elsewhere.

A possible biochemical basis for this response is suggested by the rapid increase in PAL activity after MeJA treatment. PAL catalyzes the committed entry step of the phenylpropanoid pathway and supplies precursors for downstream flavonoid biosynthesis, including flavone C-glycosides such as vitexin (Fraser and Chapple, 2011; Yao et al., 2021). The early and transient PAL induction under 75 µM MeJA, peaking within the first 12 h after elicitation, preceded the marked accumulation of TPC, TFC, and vitexin observed over the subsequent days. This temporal pattern is consistent with the view that moderate jasmonate signaling was sufficient to up-regulate the entry point (PAL) of phenylpropanoid metabolism without severely compromising cellular biosynthetic capacity. However, activation of PAL alone does not establish that the downstream flavonoid branch was coordinately induced, since the activities and transcript levels of CHS, CHI, FNS, and C-glycosyltransferase were not measured here. By analogy with other systems, MYC2-type transcription factors have been proposed to mediate jasmonate-induced expression of PAL and downstream phenylpropanoid genes (Zeng et al., 2025). The subsequent decline in PAL activity is consistent with the transient nature of jasmonate signaling and feedback regulation within the phenylpropanoid pathway (Sadeghnezhad et al., 2020). Transcriptional and enzyme-level analysis of *CHS, CHI, FNS*, and *C-glycosyltransferase* will be needed to test whether the flavonoid branch is coordinately regulated with PAL under MeJA elicitation. Phenolic and flavonoid accumulation is generally considered part of the non-enzymatic antioxidant system that limits the oxidative load generated during jasmonate signaling (Ho et al., 2020), so that the antioxidant capacity of the extracts may reflect a physiological property of the elicited tissue as well as a functional attribute of the resulting material. The antioxidant profile followed the same pattern as the phenolic and flavonoid accumulation. The 75 µM MeJA treatment produced the highest DPPH and ABTS scavenging and copper-chelating activities, suggesting that elicitation enhanced both radical-scavenging and metal-binding mechanisms. These activities are chemically consistent with the hydroxylated flavone structure of vitexin and other co-accumulated phenolic compounds, which can donate electrons or hydrogen atoms and interact with redox-active metal ions (Mira et al., 2002; Babaei et al., 2020). The concurrent rise in metal-chelating capacity is of particular physiological interest, since chelation of transition metals can suppress Fenton-type hydroxyl radical formation and would thereby complement direct radical scavenging (Mira et al., 2002). The parallel trends, in which vitexin (together with TPC and TFC) and the antioxidant activities all reached their maxima under 75 µM MeJA on Day 12, are consistent with vitexin contributing to the observed antioxidant capacity; however, because only vitexin was quantified while the extracts contain multiple phenolic and flavonoid compounds, the observed activities cannot be attributed to vitexin alone. The total activity likely reflects the combined contribution of vitexin and co-accumulated phenolics, and compound-specific fractionation or purification would be required to resolve the individual contributions. Thus, the antioxidant enhancement appears to be associated with MeJA-induced accumulation of phenylpropanoid-derived metabolites rather than a nonspecific stress response.

MeJA elicitation also improved the enzyme-inhibitory profile of *G. speciosum* PLB-derived liquid culture extracts, with 75 µM MeJA yielding the highest activities on Day 12. At the tested concentrations, the elicited extracts inhibited collagenase (55.62 ± 0.34%), elastase (42.40 ± 0.63%), and tyrosinase (36.01 ± 1.71%) to a significantly greater extent than the untreated control, with the inhibition of collagen- and elastin-degrading enzymes being more pronounced than that of tyrosinase. These findings are consistent with previous reports on *G. speciosum* extracts, in which vitexin- and orientin-rich preparations showed collagenase inhibitory activity (Yingchutrakul et al., 2021), and elicitor-treated orchid cultures showed enhanced collagenase/elastase inhibition in parallel with flavonoid accumulation (Amnuaykan et al., 2024).

Notably, this inhibition paralleled the enhanced Cu-chelating activity of the extract. As collagenase is a zinc-dependent metalloprotease and tyrosinase contains a binuclear copper center, the metal-chelating phenolics accumulating after MeJA elicitation may contribute to their inhibition by interacting with the catalytic metal ions at the enzyme active site (Crascì et al., 2017; Zolghadri et al., 2019). In contrast, elastase, a metal-independent serine protease, is more likely to be inhibited by hydrogen bonding and hydrophobic interactions (Lin et al., 2025).

While vitexin is a plausible contributor to the antioxidant and enzyme-inhibitory activities of the extracts through its flavone backbone, phenolic hydroxyl groups, 4-oxo group, and C-8 glucosyl moiety, which enable electron/hydrogen-atom donation, metal chelation, hydrogen bonding, and possible interactions with the active sites of collagenase, elastase, and tyrosinase (Babaei et al., 2020; Yan et al., 2025), vitexin represents only a fraction of the total flavonoid pool measured, and the other phenolic and flavonoid compounds present in the extracts, reflected in the TPC and TFC values, are likely to co-contribute to the observed bioactivities. Collectively, these observations suggest that MeJA-elicited *G. speciosum* PLB-derived liquid cultures may serve as a controlled *in vitro* source of flavonoid-rich, vitexin-containing extracts with potential applications as cosmetic ingredients targeting extracellular matrix protection, oxidative stress, and mild brightening.

## 5 Conclusions

In this study, PLB-derived liquid cultures of *G. speciosum* were successfully established and showed reproducible sigmoidal growth patterns suitable for elicitation studies. MeJA elicitation produced a clear growth-defense trade-off and markedly enhanced phenolic and flavonoid biosynthesis, with 75 µM identified as the most effective concentration for increasing TPC, TFC, vitexin accumulation, antioxidant capacity, and enzyme-inhibitory activities. Under these conditions, vitexin, a bioactive C-glycosylflavone previously reported to accumulate mainly in aerial tissues, reached approximately twice the level observed in untreated cultures. Its identity was confirmed by LC-ESI-QTOF-MS analysis. This vitexin accumulation was preceded by a rapid, transient increase in PAL activity, which is consistent with early up-regulation of the entry point of phenylpropanoid metabolism. Coordinated regulation of downstream flavonoid biosynthetic steps was not directly examined and remains to be verified. To our knowledge, this is the first report that MeJA elicitation enhances vitexin accumulation in *G. speciosum* cultures, offering a controlled *in vitro* alternative for laboratory-scale production of vitexin-containing extracts from this endangered CITES-listed orchid. These findings indicate that MeJA-elicited PLB-derived liquid cultures of *G. speciosum* may provide an *in vitro* platform for producing flavonoid-rich antioxidant extracts, with the observed inhibition of collagenase, elastase, and tyrosinase likely reflecting the combined contribution of vitexin and co-accumulated phenolics. Future work should focus on scaling up this culture system in bioreactors or production-scale cultivation systems to support sustained vitexin production under jasmonate elicitation.

## Data Availability

The original contributions presented in the study are included in the article/**Supplementary Material**. Further inquiries can be directed to the corresponding author.

## References

[B1] Ali M. Abbasi B. H. (2014). Thidiazuron-induced changes in biomass parameters, total phenolic content, and antioxidant activity in callus cultures of *Artemisia absinthium* L. Appl. Biochem. Biotechnol. 172, 2363–2376. doi: 10.1007/s12010-013-0663-7 24371002

[B2] Amnuaykan P. Juntrapirom S. Kanjanakawinkul W. Chaiyana W. (2024). Enhanced antioxidant, anti-aging, anti-tyrosinase, and anti-inflammatory properties of *Vanda coerulea* Griff. ex Lindl. protocorm through elicitations with chitosan. Plants 13, 1770. doi: 10.3390/plants13131770 38999610 PMC11243638

[B3] Babaei F. Moafizad A. Darvishvand Z. Mirzababaei M. Hosseinzadeh H. Nassiri-Asl M. (2020). Review of the effects of vitexin in oxidative stress-related diseases. Food Sci. Nutr. 8, 2569–2580. doi: 10.1002/fsn3.1567 32566174 PMC7300089

[B4] Cai Y. Luo Q. Sun M. Corke H. (2004). Antioxidant activity and phenolic compounds of 112 traditional Chinese medicinal plants associated with anticancer. Life Sci. 74, 2157–2184. doi: 10.1016/j.lfs.2003.09.047 14969719 PMC7126989

[B5] Chaves J. O. de Souza M. C. da Silva L. C. Lachos-Perez D. Torres-Mayanga P. C. Machado A. P. F. . (2020). Extraction of flavonoids from natural sources using modern techniques. Front. Chem. 8, 507887. doi: 10.3389/fchem.2020.507887 33102442 PMC7546908

[B6] Chusrisom J. Matmarurat G. T-Thienprasert N. P. Phonphoem W. Tongkok P. (2025). Production of bioactive compounds in *Grammatophyllum speciosum* Blume using bioreactor cultures under elicitation with sodium chloride. Plants 14, 3083. doi: 10.3390/plants14193083 41095224 PMC12526277

[B7] Crascì L. Basile L. Panico A. Puglia C. Bonina F. P. Basile P. M. . (2017). Correlating *in vitro* target-oriented screening and docking: inhibition of matrix metalloproteinases activities by flavonoids. Planta Med. 83, 901–911. doi: 10.1055/s-0043-104775 28288492

[B8] Demirci T. (2022). Determination of secondary metabolite production efficiency in *Echinacea purpurea* callus, shoot, and root *in vitro* cultures with methyl jasmonate applications. Acta Physiol. Plant 44, 128. doi: 10.1007/s11738-022-03468-630311153

[B9] Fraser C. M. Chapple C. (2011). The phenylpropanoid pathway in *Arabidopsis*. Arabidopsis Book 9, e0152. doi: 10.1199/tab.0152 22303276 PMC3268504

[B10] Geng D. Jiang M. Dong H. Wang R. Lu H. Liu W. . (2023). MeJA regulates the accumulation of baicalein and other 4′-hydroxyflavones during the hollowed root development in *Scutellaria baicalensis*. Front. Plant Sci. 13, 1067847. doi: 10.3389/fpls.2022.1067847 36684750 PMC9853287

[B11] Georgiev M. I. Weber J. Maciuk A. (2009). Bioprocessing of plant cell cultures for mass production of targeted compounds. Appl. Microbiol. Biotechnol. 83, 809–823. doi: 10.1007/s00253-009-2049-x 19488748

[B12] Ghasemzadeh A. Jaafar H. Z. Rahmat A. Ashkani S. (2015). Secondary metabolites constituents and antioxidant, anticancer and antibacterial activities of *Etlingera elatior* (Jack) R.M.Sm grown in different locations of Malaysia. BMC Complement Altern. Med. 15, 335. doi: 10.1186/s12906-015-0838-6 26399961 PMC4581154

[B13] Ho T. T. Murthy H. N. Park S. Y. (2020). Methyl jasmonate induced oxidative stress and accumulation of secondary metabolites in plant cell and organ cultures. Int. J. Mol. Sci. 21, 716. doi: 10.3390/ijms21030716 31979071 PMC7037436

[B14] Kim S. J. Sancheti S. A. Sancheti S. S. Um B. H. Yu S. M. Seo S. Y. (2010). Effect of 1,2,3,4,6-penta-O-galloyl-beta-D-glucose on elastase and hyaluronidase activities and its type II collagen expression. Acta Pol. Pharm. 67, 145–150. 20369791

[B15] Li C. Xu M. Cai X. Han Z. Si J. Chen D. (2022). Jasmonate signaling pathway modulates plant defense, growth, and their trade-offs. Int. J. Mol. Sci. 23, 3945. doi: 10.3390/ijms23073945 35409303 PMC8999811

[B16] Lin L. Yao H. Fu J. Zhang W. Li Y. Wang Y. . (2025). Elastase inhibition by natural flavonoids: mechanistic insights and potential therapeutic applications. Front. Nutr. 12, 1693869. doi: 10.3389/fnut.2025.1693869 41280381 PMC12633754

[B17] Liu N. Luo X. Tian Y. Lai D. Zhang L. Lin F. . (2019). The stereoisomeric *Bacillus subtilis* HN09 metabolite 3,4-dihydroxy-3-methyl-2-pentanone induces disease resistance in *Arabidopsis* via different signalling pathways. BMC Plant Biol. 19, 384. doi: 10.1186/s12870-019-1985-6 31488058 PMC6727425

[B18] Mira L. Fernandez M. T. Santos M. Rocha R. Florêncio M. H. Jennings K. R. (2002). Interactions of flavonoids with iron and copper ions: a mechanism for their antioxidant activity. Free Radic. Res. 36, 1199–1208. doi: 10.1080/1071576021000016463 12592672

[B19] Nhan N. H. Loc N. H. (2018). Enhancement of eurycomanone biosynthesis in cell culture of longjack (Eurycoma longifolia) by elicitor treatment. J. Plant Biotechnol. 45, 340–346. doi: 10.5010/jpb.2018.45.4.340

[B20] Rao S. R. Ravishankar G. A. (2002). Plant cell cultures: chemical factories of secondary metabolites. Biotechnol. Adv. 20, 101–153. doi: 10.1016/S0734-9750(02)00007-1 14538059

[B21] Ruiz-May E. De-la-Peña C. Galaz-Avalos R. M. Lei Z. Watson B. S. Sumner L. W. . (2011). Methyl jasmonate induces ATP biosynthesis deficiency and accumulation of proteins related to secondary metabolism in *Catharanthus roseus* (L.) G. hairy roots. Plant Cell Physiol. 52, 1401–1421. doi: 10.1093/pcp/pcr086 21727181

[B22] Rungruang R. Ratanathavorn W. Boohuad N. Selamassakul O. Kaisangsri N. (2021). Antioxidant and anti-aging enzyme activities of bioactive compounds isolated from selected Zingiberaceae plants. Agric. Nat. Resour. 55, 153–160. doi: 10.34044/j.anres.2021.55.1.20

[B23] Sadeghnezhad E. Sharifi M. Zare-Maivan H. Ahmadian Chashmi N. (2020). Time-dependent behavior of phenylpropanoid pathway in response to methyl jasmonate in *Scrophularia striata* cell cultures. Plant Cell Rep. 39, 227–243. doi: 10.1007/s00299-019-02486-y 31707473

[B24] Slinkard K. Singleton V. L. (1977). Total phenol analysis: automation and comparison with manual methods. Am. J. Enol. Vitic. 28, 49–55. doi: 10.5344/ajev.1977.28.1.49

[B25] Thamrongwatwongsa J. Chusrisom J. Katemala K. Tantasirin S. Jumnongjit P. Nateerom P. . (2024). Determination of flavonoid content in *Grammatophyllum speciosum* and *in vitro* evaluation of their anti-skin cancer and antibacterial activities. Heliyon 10, e33330. doi: 10.1016/j.heliyon.2024.e33330 39050422 PMC11266999

[B26] Vacin E. F. Went F. W. (1949). Some pH changes in nutrient solutions. Botanical Gazette 110, 605–613. doi: 10.1086/33556132539727

[B27] Wang H. Q. Jin M. Y. Paek K. Y. Piao X. C. Lian M. L. (2016). An efficient strategy for enhancement of bioactive compounds by protocorm-like body culture of Dendrobium candidum. Ind. Crops Prod. 84, 121–130. doi: 10.1016/j.indcrop.2016.02.00142574925

[B28] Woch N. Laha S. Gudipalli P. (2023). Salicylic acid and jasmonic acid induced enhanced production of total phenolics, flavonoids, and antioxidant metabolism in callus cultures of *Givotia moluccana* (L.) Sreem. In Vitro Cell. Dev. Biol. Plant 59, 227–248. doi: 10.1007/s11627-023-10335-730311153

[B29] Yan W. Cheng J. Xu B. (2025). Dietary flavonoids vitexin and isovitexin: new insights into their functional roles in human health and disease prevention. Int. J. Mol. Sci. 26, 6997. doi: 10.3390/ijms26146997 40725244 PMC12296012

[B30] Yao T. Feng K. Xie M. Barros J. Tschaplinski T. J. Tuskan G. A. . (2021). Phylogenetic occurrence of the phenylpropanoid pathway and lignin biosynthesis in plants. Front. Plant Sci. 12, 704697. doi: 10.3389/fpls.2021.704697 34484267 PMC8416159

[B31] Yingchutrakul Y. Sittisaree W. Mahatnirunkul T. Chomtong T. Tulyananda T. Krobthong S. (2021). Cosmeceutical potentials of *Grammatophyllum speciosum* extracts: anti-inflammations and anti-collagenase activities with phytochemical profile analysis using an untargeted metabolomics approach. Cosmetics 8, 116. doi: 10.3390/cosmetics804011630654563

[B32] Zeng T. Su H. Wang M. He J. Gu L. Wang H. . (2025). The role of MYC2 transcription factors in plant secondary metabolism and stress response mechanisms. Plants 14, 1255. doi: 10.3390/plants14081255 40284143 PMC12030399

[B33] Zhang A. Fu L. Zuo F. Qian L. (2022). HPLC analysis of vitexin and isovitexin content changes during mung bean germination. J. Food Meas Charact 16, 3302–3309. doi: 10.1007/s11694-022-01376-430311153

[B34] Zhong L. Liu Y. Xiong B. Chen L. Zhang Y. Li C. (2019). Optimization of Ultrasound‐Assisted Extraction of Total Flavonoids from Dendranthema indicum var. aromaticum by Response Surface Methodology. J. Anal. Methods Chem. 2019, 1648782. doi: 10.1155/2019/1648782 31396433 PMC6664694

[B35] Zhu X. Chen J. Qiu K. Kuai B. (2017). Phytohormone and light regulation of chlorophyll degradation. Front. Plant Sci. 8, 1911. doi: 10.3389/fpls.2017.01911 29163624 PMC5681529

[B36] Zolghadri S. Bahrami A. Hassan Khan M. T. Munoz-Munoz J. Garcia-Molina F. Garcia-Canovas F. . (2019). A comprehensive review on tyrosinase inhibitors. J. Enzyme Inhib Med. Chem. 34, 279–309. doi: 10.1080/14756366.2018.1545767 30734608 PMC6327992

