# Supplementary material for: Methyl jasmonate elicitation increases vitexin production in protocorm-like body-derived cultures of Grammatophyllum speciosum Blume
Source: Front Plant Sci. 2026 Oct 28;17:1915401. doi: 10.3389/fpls.2026.1915401 (PMC13753958; doi:10.3389/fpls.2026.1915401)
Supplement: Supplementary file 1 [file SupplementaryFile1.pdf]

Supplementary Material

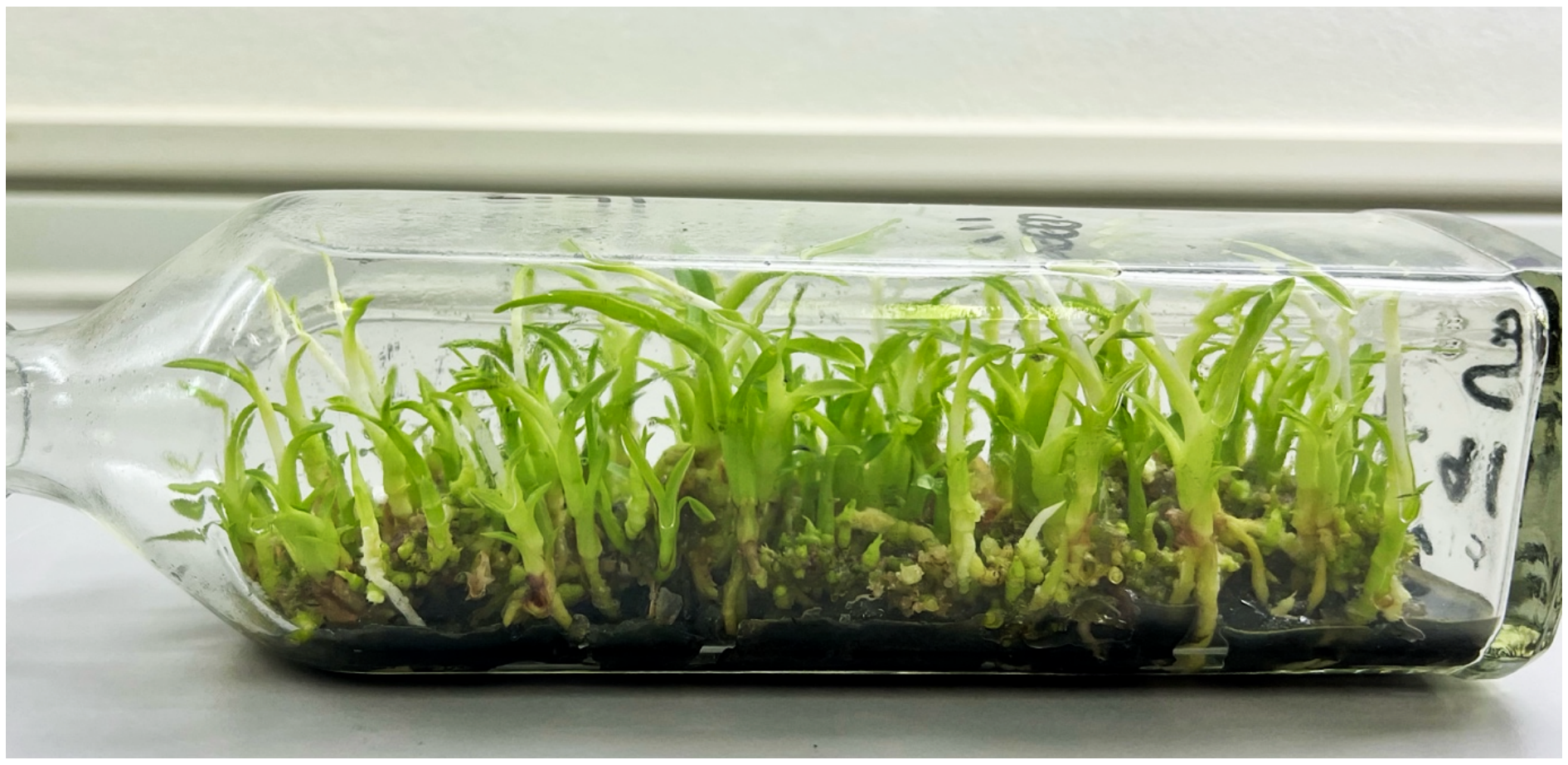

Supplementary Figure 1. PLB culture on solid VW medium, showing PLBs and developing plantlets.

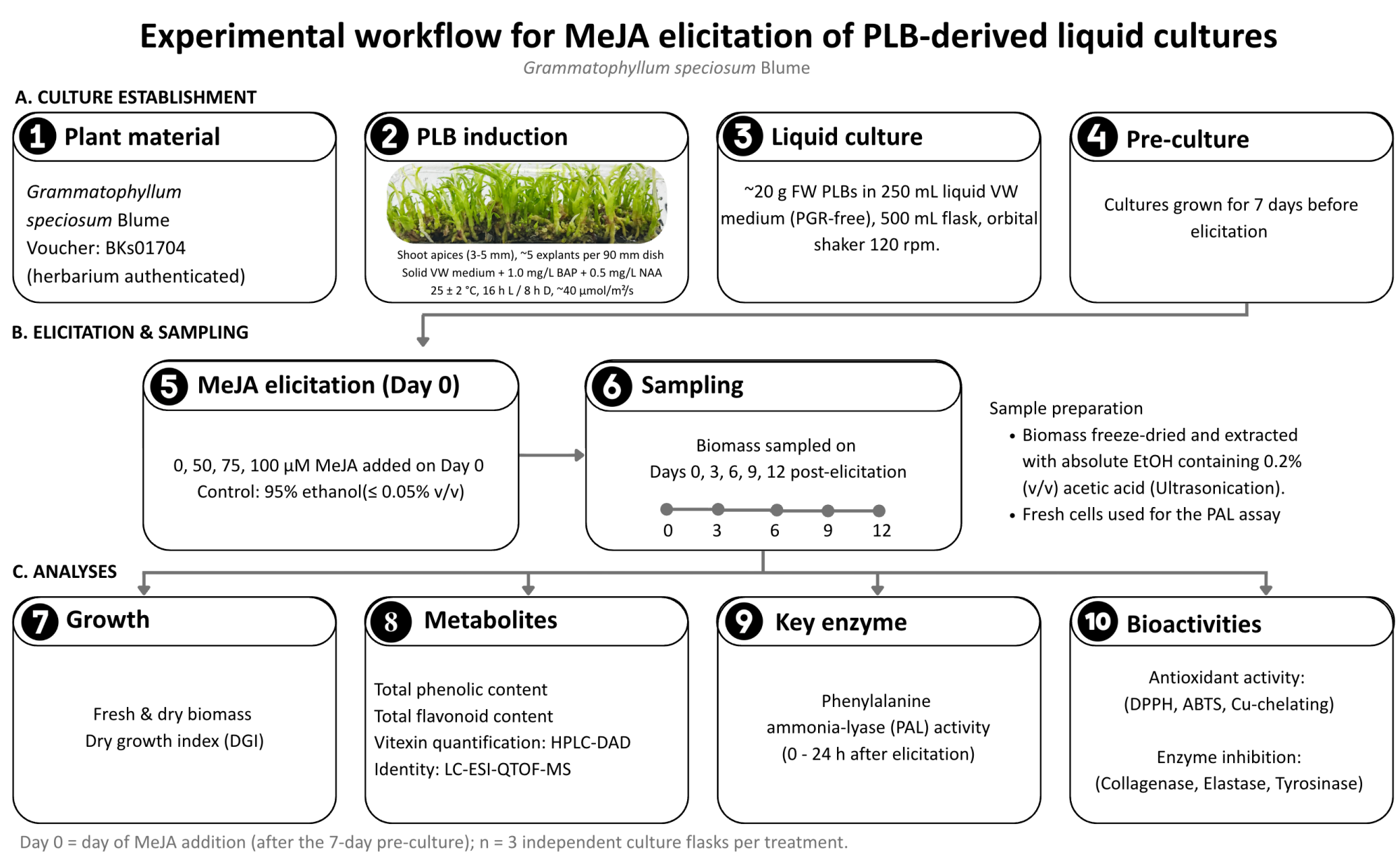

Supplementary Figure 2. Experimental workflow for *G. speciosum* PLB-derived liquid culture and MeJA elicitation, comprising (A) culture establishment, with a representative photograph of PLBs on solid VW medium, (B) MeJA elicitation (0, 50, 75, and 100 μM) and sampling on Days 0-12, and (C) the analyses performed. Day 0 denotes the day of MeJA addition, after the 7-day pre-culture; *n = 3* independent culture flasks per treatment.

Supplementary Table 1. Analytical characteristics of the HPLC–DAD method used for vitexin quantification.

Chromatographic conditions: RP-HPLC ODS-3 C18 column (4.6 × 150 mm, 5 µm) at 35 °C; mobile phase, acetonitrile (A) and 0.2% (v/v) aqueous acetic acid (B) under gradient elution; flow rate 1.0 mL min^{-1}; injection volume 5 µL; diode-array detection at 336 nm.

Parameter	Value
Retention time, t_r (min)	16.212 ± 0.022
Detection wavelength (nm)	336
Regression equation	y = 22.3024x + 0.510
Linearity range (µg mL^{-1})	1 - 100
Coefficient of determination (R^2)	0.9971

Values are shown for the HPLC-DAD method adapted from Zhang et al. (2022). LOD, LOQ, recovery, intra- and inter-day precision, chromatographic resolution, and peak purity were not experimentally determined in the present study, and dedicated method validation is identified as follow-up work. Compound identity was independently confirmed by LC-ESI-QTOF-MS.

Supplementary Table 2. Vitexin content and yield per flask in *G. speciosum* PLB-derived liquid cultures on Day 12 after MeJA elicitation.

MeJA (µM)	Vitexin content (µg/g CDW)	Vitexin yield (µg/flask)
0 (control)	208.33 ± 2.69 ^{a}	15.49 ± 0.21
50	302.14 ± 1.98 ^{b}	21.93 ± 0.27
75	420.05 ± 9.96 ^{d}	30.45 ± 0.75
100	326.22 ± 3.01 ^{c}	23.42 ± 0.22

Values are means ± SD from three independent culture flasks (n = 3). Different lowercase letters indicate significant differences in vitexin content among MeJA concentrations ($p < 0.05$, Tukey's HSD; one-way ANOVA $F(3, 8) = 760.44$, $p < 0.001$; letters assigned in ascending order, a = lowest).

Supplementary Table 3. ANOVA results for the effects of MeJA concentration (and cultivation day/time where applicable) on the responses of *G. speciosum* PLB-derived liquid cultures.

Response variable	Source of variation	df	F	p-value
**Dry growth index (DGI) ***	MeJA concentration	3, 32	44.24	< 0.001
	Cultivation day	3, 32	194.60	< 0.001
	MeJA × Day	9, 32	3.79	= 0.002
**Total phenolic content (TPC)**	MeJA concentration	3, 40	600.20	< 0.001
	Cultivation day	4, 40	1093.80	< 0.001
	MeJA × Day	12, 40	95.00	< 0.001
**Total flavonoid content (TFC)**	MeJA concentration	3, 40	287.14	< 0.001
	Cultivation day	4, 40	386.07	< 0.001
	MeJA × Day	12, 40	34.55	< 0.001
**Vitexin content ****	MeJA concentration	3, 8	760.44	< 0.001
**PAL activity *****	MeJA concentration	3, 40	371.90	< 0.001
	Time (h)	4, 40	247.38	< 0.001
	MeJA × Time	12, 40	46.58	< 0.001
**DPPH radical scavenging**	MeJA concentration	3, 40	595.44	< 0.001
	Cultivation day	4, 40	722.79	< 0.001
	MeJA × Day	12, 40	46.87	< 0.001
**ABTS radical scavenging**	MeJA concentration	3, 40	2734.03	< 0.001
	Cultivation day	4, 40	2116.94	< 0.001
	MeJA × Day	12, 40	180.10	< 0.001
**Copper chelating activity**	MeJA concentration	3, 40	220.23	< 0.001
	Cultivation day	4, 40	184.25	< 0.001
	MeJA × Day	12, 40	23.94	< 0.001
**Anti-collagenase activity**	MeJA concentration	3, 40	86.85	< 0.001
	Cultivation day	4, 40	147.80	< 0.001
	MeJA × Day	12, 40	10.61	< 0.001
**Anti-elastase activity**	MeJA concentration	3, 40	236.81	< 0.001
	Cultivation day	4, 40	523.55	< 0.001
	MeJA × Day	12, 40	28.30	< 0.001
**Anti-tyrosinase activity**	MeJA concentration	3, 40	142.65	< 0.001
	Cultivation day	4, 40	434.57	< 0.001
	MeJA × Day	12, 40	13.86	< 0.001

Two-way ANOVA (or one-way ANOVA for vitexin content) was performed on data from three independent biological replicates (n = 3) per treatment and cultivation day. Analyses were carried out in SPSS version 28 (IBM Corp., Armonk, NY, USA) using Type III sum of squares. Normality and homogeneity of variance were verified using Shapiro–Wilk and Levene's tests, respectively. df values are presented as (numerator, denominator).

* DGI was measured on Days 3, 6, 9, and 12 only, since DGI is defined as zero at Day 0 by design; this yields four rather than five-day levels, giving df error = 32.

** Vitexin content was quantified only at the endpoint (Day 12); therefore, the effect of MeJA concentration was assessed by one-way ANOVA.

*** PAL activity was measured at 0, 6, 12, 18, and 24 h after MeJA addition rather than over the 12-day cultivation period.
